# Integrated metabolomic and metagenomic strategies shed light on interactions among planting environments, rhizosphere microbiota, and metabolites of tobacco in Yunnan, China

**DOI:** 10.3389/fmicb.2024.1386150

**Published:** 2024-05-09

**Authors:** Rentao Liao, Zhengjie Liu, Wenhua Dongchen, Xiaopeng Deng, Erdeng Ma, Nazer Manzoor, Chun Lin, Shaosong Zhou, Wenjie Tong, Min Zhou, Junying Li, Zichao Mao

**Affiliations:** ^1^Yunnan Academy of Tobacco Agriculture Sciences, Kunming, China; ^2^College of Agronomy and Biotechnology, Yunnan Agricultural University (YNAU), Kunming, China; ^3^Institute of Improvement and Utilization of Characteristic Resource Plants, Kunming, China; ^4^Agricultural Environmental Resources Research Institute, Yunnan Academy of Agricultural Sciences, Kunming, China; ^5^The Laboratory for Crop Production and Intelligent Agriculture, YNAU, Kunming, China

**Keywords:** planting environment, metabolomics, metagenomics, tobacco, interactions

## Abstract

Changes in climatic factors and rhizosphere microbiota led plants to adjust their metabolic strategies for survival under adverse environmental conditions. Changes in plant metabolites can mediate crop growth and development and interact with rhizosphere microbiota of the plant rhizosphere. To understand the interactions among environmental factors, rhizosphere microbiota, and metabolites of tobacco, a study was conducted by using integrated metagenomic and metabolomic strategies at four typical representative tobacco planting sites in Yunnan, China. The results showed that the agronomical and biochemical traits were significantly affected by temperature, precipitation (PREP), soil pH, and altitude. Correlation analyses revealed a significant positive correlation of temperature with length, width, and area of the leaf, while PREP correlated with plant height and effective leaf numbers. Furthermore, total sugar and reducing sugar contents of baked leaves were significantly higher, while the total nitrogen and total alkaloid levels were lower in tobacco leaves at site with low PREP. A total of 770 metabolites were detected with the highest number of different abundant metabolites (DMs) at Chuxiong (CX) with low PREP as compared to the other three sites, in which secondary metabolites were more abundant in both leaves and roots of tobacco. A total of 8,479 species, belonging to 2,094 genera with 420 individual bins (including 13 higher-quality bins) harboring 851,209 CDSs were detected. The phyla levels of microorganisms such as Euryarchaeota, Myxococcota, and Deinococcota were significantly enriched at the CX site, while Pseudomonadota was enriched at the high-temperature site with good PREP. The correlation analyses showed that the metabolic compounds in low-PREP site samples were positively correlated with *Diaminobutyricimonas*, *Nissabacter*, *Alloactinosynnema,* and *Catellatospora* and negatively correlated with *Amniculibacterium*, *Nordella*, *Noviherbaspirillum,* and *Limnobacter,* suggesting that the recruitment of *Diaminobutyricimonas*, *Nissabacter*, *Alloactinosynnema,* and *Catellatospora* in the rhizosphere induces the production and accumulation of secondary metabolites (SMs) (e.g., nitrogen compounds, terpenoids, and phenolics) for increasing drought tolerance with an unknown mechanism. The results of this study may promote the production and application of microbial fertilizers and agents such as *Diaminobutyricimonas* and *Alloactinosynnema* to assemble synthetic microbiota community or using their gene resources for better cultivation of tobacco as well as other crops in drought environments.

## 1 Introduction

Climatic factors and soil conditions correlate to rhizosphere microbiota and finally influence crop growth and yield (Bacenetti et al., 2015; Vogel et al., 2019; Klein et al., 2022). Extreme biotic and abiotic stress factors cause plant structural and functional damage, result in reactive oxygen species (ROS) burst (Cruz de Carvalho, 2008) and changes in the antioxidants and related enzymatic activities (Abid et al., 2018; Laxa et al., 2019). The plants also change their metabolic strategies to accumulate various protective metabolites for survival during stressful experiences (Capell et al., 2004; Quan et al., 2004). Fluctuations in plant metabolites can shape the microbiota of the plant root surface, especially the rhizosphere (Sohn et al., 2022), and the rhizosphere microbial community plays an important role in regulating growth and development of plant hosts, including enhancing host stress tolerance, regulating host immune processes, improving host environmental adaptability for mineral absorption, and changing metabolite profiles (Korenblum et al., 2020; Petipas et al., 2021).

Tobacco (*Nicotiana tabacum* L.) is a cash crop as well as an important model plant that plays a significant role in both basic and applied research studies (Jurečková et al., 2017). Biosynthesis and accumulation of tobacco metabolites are significantly influenced by environmental conditions, especially climatic factors, its soil nutrition, and rhizosphere microorganisms (Li et al., 2023). Previous studies have shown that water condition is closely related to the formation and accumulation of different metabolites in the tobacco cultivar “Yun87” (Tang et al., 2020).

Currently, over 2,500 metabolites have been identified in tobacco (Jassbi et al., 2017). Among them, various secondary metabolites (SMs) play an important ecological and protective role in increasing plant stress tolerance (Severson et al., 1991; Baldwin, 1999). The SMs of tobacco were generally classified into several categories, including terpenoids (e.g., plant volatiles, triterpenes, and steroidal saponins), phenolics (e.g., phenolic acids, flavonoids, lignins, and tannins), alkaloids (e.g., nicotine), and sulfur-containing compounds (e.g., glucosinolates) (Jassbi et al., 2017). Terpenoids are important for tobacco odor (Loughrin et al., 1990; Schlotzhauer et al., 1995; Raguso et al., 2006), serving as effective insecticides (Hummelbrunner and Isman, 2001) and antibacterial agents (Taniguchi et al., 2014); for example, terpenoids are widely involved in physiological activities and play a wide range of ecological roles, such as defense (Kessler and Baldwin, 2001) and gene expression regulation [e.g., gibberellic acids (GAs), abscisic acid (ABA), and brassinosteroids (BRs)] (Yang et al., 2014). Phenolic compounds are mostly abundant in plants with effective antioxidants (Ru et al., 2012), playing important roles in inhibiting microbial growth (Wang et al., 2008), with antiviral as well as anti-radiational effects (Miao et al., 2015). Alkaloids are widely distributed in tobacco plants as effective defensive metabolites against herbivores or competing with other environmental plants through allelopathy (Wink, 1988).

Research studies in *Arabidopsis thaliana* showed that metabolites of root exudates including both primary metabolites (PMs) and SMs of plants account for ~10% of photosynthetical carbon and ~15% of total plant nitrogen compounds (Walker et al., 2003). In addition, the root exudates can significantly shape the diversities and mediate mutual interactions between plants and microbes by (1) the rhizosphere microbial communities obtaining sources of both nitrogen and carbon from root exudates for survival or suppressing microbial processes by plant antibiotic metabolites, (2) the beneficial microbiota providing plant bioavailable mineral nutrients, plant regulatory components (e.g., phytohormones), or suppressing the pathogens by means of ecological competition or synthesizing pathogen-suppressing chemicals, and (3) the rhizosphere pathogens or harmful microbiota inducing plant systemic resistant reactions or leading to damage of plant hosts. Pioneering research studies showed that (Neal et al., 2012) the composition of root exudates varies not only among different plant species but also among the same species with changed ecological environments (e.g., extreme temperature, drought, and UV radiation). The benzoxazinoids (one of indole-derived metabolites) can recruit a growth-promoting *Pseudomonas putida* to the root surface of maize, while in *A. thaliana* (Voges et al., 2019), the abundance of a *Pseudomonas* strain in its rhizosphere is significantly higher in the coumarin (a phenolic compound)-deficient mutant of *A. thaliana* compared with its wild-type. Huang et al. (2019) showed that triterpenes also play a key role in modulating the *A. thaliana* rhizosphere microbiota. Except the SMs, other PMs (e.g., amino acids and sugars) also drive pattern changes in the rhizosphere microbiota; simultaneously, the rhizosphere microorganisms encode multiple membrane transporters to facilitate transport of plant exudates into bacterial cells for a mutual interaction between the plant and its rhizosphere microbiome (Mauchline et al., 2006).

Yunnan located in southwest of China has geographic characteristics of a low-latitude plateau, which makes it an ecologically diverse environment for growing various crops. Moreover, about half of tobacco produced in China is planted in Yunnan, and planting and harvesting of tobacco leaves have been the major source of income for millions of farmers, especially in poor areas of Yunnan (Teh-wei et al., 2010). To understand the interaction among the rhizosphere bacteriomes of tobacco, Cao et al. (2023) conducted amplicon sequencing of 16S rDNA to investigate the dynamic changes in soil bacteria between the healthy and root-knot nematode-infected tobacco plants, with similar strategies. Zhou et al. (2023) studied different intercropping system-modulated soil–bacterial communities that shaped the growth and development status of tobacco. Zhao et al. (2023) studied the correlations between differences in soil physicochemical properties and diversified rhizosphere microbiota during different transplanting stages of tobacco at two sites of Linxiang district, Lincang city, Yunnan. Currently, there is no report on direct interactions between tobacco metabolites and its rhizosphere microbiota; thus, this study uses the major tobacco variety of Yunnan “Yun87” as the test cultivar in four experimental sites (i.e., Chuxiong City (CX) in Chuxiong Yi Autonomous Prefecture, Jianshui County (JS) in Honghe Hani Yi Autonomous Prefecture, Xiangyun County (XY) in Dali Bai Autonomous Prefecture, and Zhanyi District (ZY) in Qujing City), which are typical representative sites of tobacco-producing areas in Yunnan to conduct this experiment.

As there are difficulties in collecting root exudates (normally collected by water solution cultivation, or the potted plant root surface attached or covered by a special filter paper for collection) from natural soil cultivating conditions in the field, so integrated metagenomic and metabolomic strategies were chosen to conduct analyses of (1) the rhizosphere microorganism diversities of planting tobacco rhizosphere soil in Yunnan, (2) the effect of climatic factors on the growth and development, the rhizosphere microbiota, and the metabolites of tobacco cultivar of “Yun87,” and (3) correlations between soil rhizosphere microorganisms and plant metabolites, intended to elucidate interactions between plant metabolites and its rhizosphere microorganisms as well as providing a reference to choose the beneficial microbiota for assembling synthetic microorganism communities better cultivation of tobacco or other important crops.

## 2 Materials and methods

### 2.1 Plant materials and cultivation methods

“Yun87” was chosen as the tested tobacco variety, and the planting sites were Chuxiong City (CX, 24° 94′N, 101° 49′E, altitude 1846 m), Jianshui County (JS, 23° 31′N, 102° 45′E, altitude of 1,414 m), Xiangyun County (XY, 25° 25′N, 100° 46′E, altitude 1,943 m), and Zhanyi District (ZY, 25° 41′N, 103° 40′E, altitude 2,009 m). The agricultural performance of the tobacco variety was uniform at all four planting sites in 2022. According to the methods of Chinese soil taxonomy, the planting soil types in JS, XY, and ZY are typical red soil in which maize was planted in the previous year (2021), while CX had paddy soil in which paddy rice was planted in 2021. The average pH, soil organic matter (SOM), alkali-hydrolyzed nitrogen (AN), available phosphorus (AP), available potassium (AK), and soil chlorine (SCL) were determined and shown in Supplementary Table S1. Healthy tobacco seedlings with a height of 5–7 cm and having 4–5 leaves were prepared by floating germination and cultivation from 4th of March 2022. The transplantation of seedlings under plastic films was conducted in a 300 m^3^ test plot with three replications. The spacing of the planted tobacco was set at 60 cm × 120 cm, which resulted in ~16,725 plants per hectare (Ha). The fertilizer (N:P_2_O_5_:K_2_O = 15:18:15) was applied with a dose of 60 kg/Ha in planting holes before transplantation of seedlings, on May 13th after planting for ~2 weeks, and the same type of fertilizer (N:P_2_O_5_:K_2_O = 15:18:15) with a dose of 240 kg/Ha was dissolved in water and directly applied to the root area of the planted tobacco. After 2 weeks, 255 kg/Ha dose of nitrogen and potassium (N-P) fertilizer (N:P_2_O_5_:K_2_O = 12.5:0:33.5) plus 75 kg/Ha K_2_SO_4_ were applied on May 26th, and then 90 kg/Ha (N:P_2_O_5_:K_2_O = 15:18:15), 180 kg/Ha N-P fertilizer (N:P_2_O_5_:K_2_O = 12.5:0:33.5), and 255 kg/Ha K_2_SO_4_ were applied on June 7th; simultaneously, the covered films were removed for continuing cultivation until leaf harvesting. The number of final left leaves for harvesting per plant was 18–20, while the height of tobacco plants was 95–110 cm; the apical meristems were removed, and the mature leaves were collected three times, followed by the leaves being baked using an intelligent baking method.

### 2.2 Soil sample collection and analysis

By using the “S” type sampling method, before the transplantation of seedlings, five of 0–20 cm top soil samples from each planting sites of CX, JS, XY, and ZY were collected with three replications. For each soil sample, 10 subsamples were collected and pooled to make up a final 1-kg top soil sample. According to Yunnan local standard methods of NY/T1377-2007, NY/T1121.6-2006, LY/T1228-2015, NY/T1121.7-2014, NY/T889-2004, and NY/T1378-2007, the soil pH, SOM, AN, AP, AK, and SCL were determined, respectively. To sample rhizosphere soil for metagenomic analyses, each soil sample (2–3 g with three replications) was collected from root surfaces of healthy tobacco plants developed into the productive phase with 1–2 opened flower (s) from the four planting sites (CX, JS, XY, and ZY). In detail, the upper 2–3 cm layer of soil was removed, then tobacco plants were uprooted (five plants per sample), and rhizosphere soil samples were collected from tobacco root surfaces with removable visible root systems.

### 2.3 Tobacco sampling and determination of their agronomic traits and the baked tobacco leaf chemical analyses

The five-point sampling method was used in different planting sites, and for each sampling point, 10 tobacco plants were selected, after measuring their agronomic traits such as plant height, leaf length, leaf width, effective leaf numbers, and leaf area (calculated with formula of leaf length × leaf width × 0.6345). When the tobacco was developed into the productive phase with 1–2 opened flowers, the largest leaf in the middle of the tobacco plant and the same plant tobacco root system (removing soil by washing with distilled water) were frozen in liquid nitrogen, then stored at −80°C, or directly used for metabolomic analyses. One kilogram of middle-baked tobacco leaves was collected for total sugars (TS), reducing sugars (RS), total nitrogen (TN), and total alkaloid (TA) analyses with three biological replications using the standard determining methods of DB12/T 847-2018, YC_T 159-2019, YC_T 33-1996, and YC/T 468-2021, respectively.

### 2.4 Tobacco metabolite analysis

According to the reported method of Li et al. (2024), the metabolic analyses of both leaves and roots were performed. Briefly, about 150 mg of each sample was ground in 1 mL extraction solution (methanol: water, 7:3, v/v) for 10 min at 4°C, then the mixture was centrifuged at 13,000 × g for 10 min to collect supernatants (three times), and the collected supernatants were filtered with a 0.22 μm membrane for LC-MS/MS analyses. The following analytical LC-MS/MS station was used: ACQUITY UPLC I-Class Plus (Waters, United States) series QTRAP6500 plus high-sensitivity mass spectrometer (SCIEX, United States) equipped with the Waters column of HSS T3 (2.1 mm × 10 cm, 1.8 μm) for metabolic separation detection. The analytic mobile system consists of solvent A (water with 0.1% formic acid) and solvent B (acetonitrile with 0.1% formic acid). The separation was performed with a linear gradient elution program starting from 95% A, 5% B to 5% A, 95% B within 10 min and then a composition of 5% A, 95% B kept for 1 min. Subsequently, a composition of 95% A, 5% B was adjusted within 0.5 min and kept for 5 min with an injection volume of 2 μL filtered supernatants. We used quantitative software skyline (v.21.1.0.146), combined with the BGI^1^ widely targeted metabolic standard database, for metabolite identification and quantitative analyses. After obtaining metabolite structures and their quantitative values, PLS-DA analysis was performed using the R package of ropls (v1.28.2), and differentially abundant metabolites (DMs) between the two planting sites were screened with absolute log_2_FC ≥1 and VIP >1 with the web resource of Metware Cloud^2^ as well as Bioloader,^3^ respectively. The abundance of DMs was visualized by the R package of pheatmap.^4^

### 2.5 Tobacco rhizosphere microbiota diversities, their gene annotation, and abundance analyses

Total genomic DNAs were extracted from ~1 g of the rhizosphere soil per sample with the FastDNA^™^ Spin Kit for Soil (MP Biomedicals, United States) following the manufacturer’s protocol. The qualified DNA samples were sent to BGI for library preparation and sequencing on the Illumina NovaSeq 6000 platform (Illumina, United States) to generate ~10 Gb DNA sequencing raw data per sample with three replications. The quality of raw data was checked with fastQC (v0.11.9, https://github.com/csf-ngs/fastqc/tree/master/fastqc-csf), then the low-quality reads were filtered, and the adapter with script of fastp (v0.11.9, https://github.com/OpenGene/fastp) was removed using default parameters to get clean reads. For removing tobacco genomic DNA contamination, bowtie2 (v2.51, https://github.com/BenLangmead/bowtie2) was used for mapping the obtained clean fastq reads to the available tobacco genome downloaded from the Solanaceae Genomics Network^5^ (Edwards et al., 2017) to obtain the bam file, and then samtools (v1.13, https://github.com/samtools/samtools) was further used to filter reads with a flag value of 12 (meaning reads unmapped or mate unmapped to the tobacco genome) to get clean DNA fastq data for soil microbiota analyses. The megahit (v1.2.9, https://github.com/voutcn/megahit) was used for metagenomic assembling. In order to identify individual genomes from microbiota samples, the megahit-assembled DNA contigs were further separated into bins with MaxBin (v2.2.7) (Wu and Singer, 2021), followed by CheckM (v1.1.3 https://github.com/Ecogenomics/CheckM) and dRep (v3.2.2, https://github.com/MrOlm/drep) to check the quality and remove the duplicated bins to finally obtain high-quality individual bin (Hbins) genomes. The DNA of obtained bins and Hbins was classified or identified by GTDB-tk (v2.3.0, https://github.com/Ecogenomics/GTDBTk/) with release database of 214 (GTDB, https://gtdb.ecogenomic.org/). Metagenome structural genes (CDSs) were annotated by popular pipelines of prokka (v1.14.6) (Seemann, 2014) based on the script of prodigal (v2.6.3, https://github.com/hyattpd/Prodigal), and then redundant CDSs were removed by cdhit,^6^ and finally the CDS functions were annotated by eggnog-mapper,^7^ and homologous searching with the Uniref90 database^8^ was conducted by diamond (v 0.9.14, https://github.com/python-diamond/Diamond). The R packages of DEseq2 (V1.36.0) and clusterProfiler (v4.4.4) were used for differentially abundant genes (DAGs) as well GO and KEGG enrichment analyses for DAGs of microbiota from four tobacco planting sites after their clean fastq reads have been mapped to CDSs by salmon (v1.1.0, https://github.com/SALMON-TDDFT/SALMON).

For further analyses of diversity, abundance, and taxonomy of rhizosphere microbiota, the k-mer strategy was used by Kraken2 (v2.1.2), followed by Bracken (v2.6.2) (Lu et al., 2022) based on salmon scripts with default parameters. The analyses of alpha (including chao1 value, obtained operational taxonomic unit (OTU), the Simpson value and Shannon values) and beta diversities in microbiota were performed through R packages of vegan (v2.6.4) and phyloseq (v1.40). The R packages of ggcor (v0.9.8.1) and vegan (v2.6.4) were used to determine the correlations among metabolites, planting environmental factors, the abundance of metagenomic genes, and abundance of microbiota by the Mantel test and canonical correlation analyses (CCA), and LEfSe (v1.1.2) and varSelRF (v0.7.8) were used for screening of key differentially abundant species (DASs). The differentially abundant genes (DAGs) and the abundance of microbiota were visualized by the R package of pheatmap (v1.0.12). Partial least squares path modeling (PLS-PM) was used for model construction with the R package of plspm (v0.5.1).

### 2.6 Statistical analyses, chemical drawings, and integration of figures

The statistical analyses were conducted by the R package of rstatix (v0.72, https://github.com/kassambara/rstatix), and the significance differences were filtered by *p* < 0.05 or extremely significant differences by *p* < 0.01. Chemical structures were drawn by ChemDraw (v 20.0), and Adobe Illustrator (v26.4.1) was used for integrating or beautifying figures.

## 3 Results and analyses

### 3.1 Climate differences in four geographical regions

**Figure 1:**
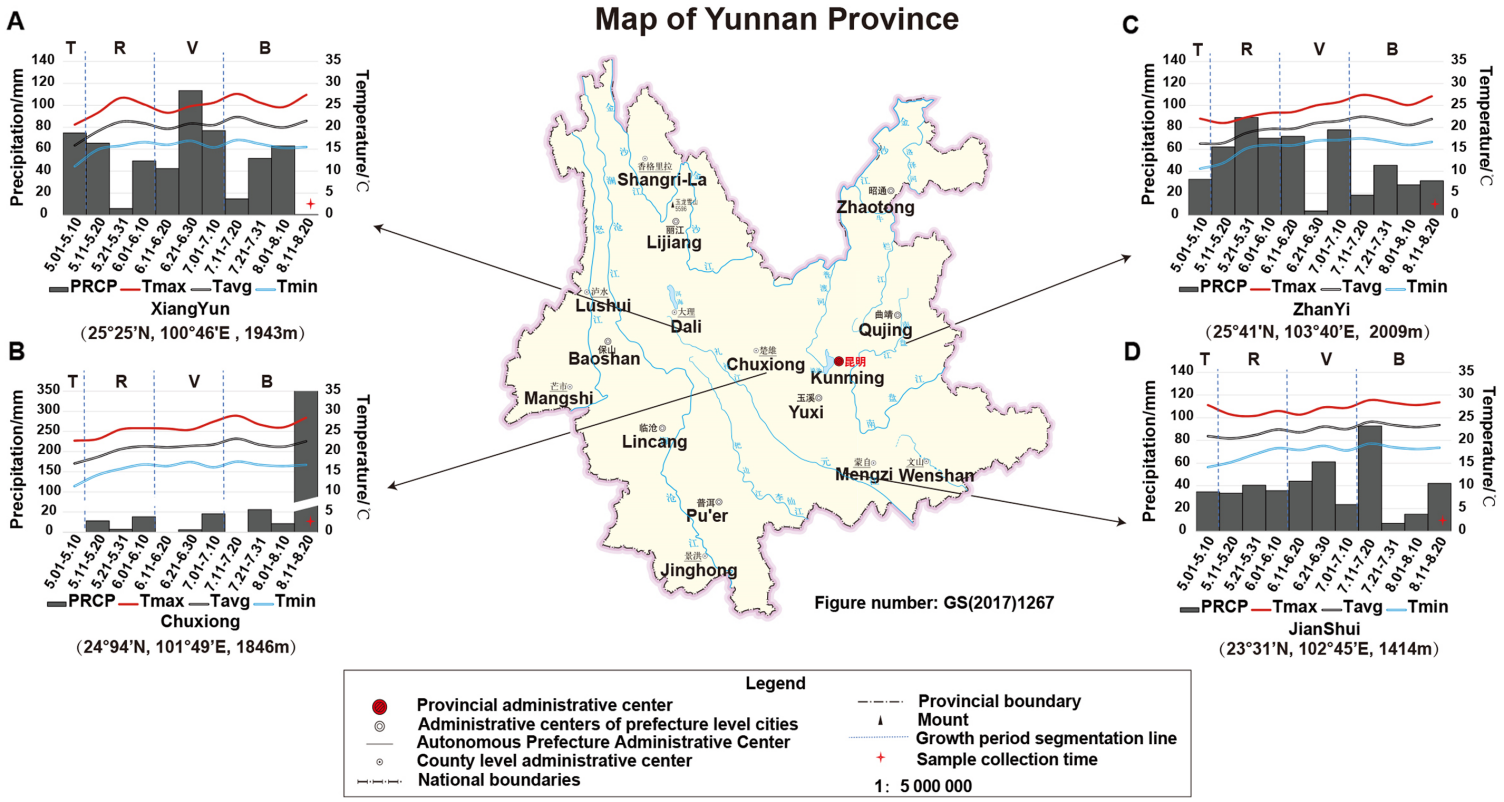
The location and climatic condition of four planting sites of XY **(A)**, CX **(B)**, ZY **(C)**, and JS **(D)**. Tmax: maximum temperature, Tavg: average temperature, Tmin: minimum temperature, and PREP: precipitation of 10 day’s average of the planting period of four planting sites in Yunnan; T, R, V, and B: transitioning period, rosette period, vigorous growing period, and budding and maturation period during tobacco growth, respectively. The planting site’s latitude, longitude, and altitude are shown below of each figure, respectively.

The growth period of tobacco in Yunnan, China, is generally divided into four periods: transplanting period (T) from May 1st to May 9th, rosette period (R) from May 11th to June 10th, vigorous growing period (V) from June 11th to July 10th, and budding and maturation period (B) from July 11th to early September. In this study, the climatic data of each growing period in the four planting sites (e.g., temperature and precipitation) were obtained from the web source^9^ or collected from local climatic station. The data showed that (Figure 1) during the tobacco T period, the PREP and air temperature in JS were higher than that in the other three sites. However, there was nearly no PREP at T period in CX (therefore, one-time irrigation was applied to CX tobacco by adding 1.5 L of water per plant on May 10th to ensure the survival of the CX tobaccos), while the accumulated PREP in other sites was greater than 30 mm, especially in XY, in which it reached 74.78 mm. During the tobacco R period, the temperature in ZY was lower due to the high PREP and location more to the north with higher altitude (25° 41′N, 103° 40′E, altitude 2,009 m). During the tobacco V period, the temperatures in JS and CX were higher than those in XY and ZY, but the average PREP in CX was lower than that in ZY, JS, and XY. During the tobacco B period, the CX site experienced heavy rainfall on August 15th, with a daily PREP of 341.14 mm; however, during this period, the temperature in both CX and JS was still higher than that in XY and ZY. From the perspective of the entire growth period of tobacco, less PREP was the most prominent environmental factor in CX leading to both high temperature and drought stress for the growth and development of CX tobaccos (Figure 1).

### 3.2 The agronomic characteristics of tobacco

Data of agronomic traits at the B period of tobacco with 1–2 opened flowers in all planting sites were collected, and statistical analyses were conducted focusing on plant height, effective leaf numbers, leaf length, leaf width, and leaf area of tobacco in the four planting sites. As shown in Table 1, it was found that there were significant differences in plant height among the four planting sites, with XY > JS > ZY > CX. There was no significant difference in the numbers of effective leaf between ZY and CX, with XY > JS > ZY & CX. There were significant differences in leaf length among the four sites, with JS > XY > ZY > CX. There was no significant difference in leaf width between CX and XY, with JS > XY & CX > ZY. There were significant differences in the leaf area among four planting sites, with JS > XY > CX > ZY (Table 1). For quality evaluation of baked commercial leaves at the four planting sites, the TS, RS, TN, and TA were determined, and the results showed that the TS and RS in CX and XY had no significant differences, while JS and ZY also had no differences, whereas CX and XY > JS and ZY. However, different patterns found in TN and TA had opposite results of both TS and RS, with CX and XY < JS and ZY (Table 2).

**Table 1:** Agronomic traits of tobacco during the budding period in four planting sites.

Sites	Plant height (cm)	Effective number of leaves	Leaf Length (cm)	Leaf width (cm)	Leaf area (cm^2^)
CX	105.28 ± 3.637 dC	16.20 ± 0.789 cC	55.25 ± 4.323 dC	26.70 ± 2.555 bB	938.15 ± 132.638 cC
JS	125.77 ± 8.484 bB	19.77 ± 0.898 bB	72.83 ± 4.481 aA	29.00 ± 2.364 aA	1344.46 ± 172.793 aA
XY	134.63 ± 9.313 aA	21.20 ± 2.024 aA	67.03 ± 3.439 bB	27.15 ± 2.808 bAB	1157.07 ± 151.944 bB
ZY	119.97 ± 5.372 cB	17.00 ± 1.486 cC	57.90 ± 2.881 cC	19.30 ± 1.512 cC	709.77 ± 72.542 dD

**Table 2:** The chemical composition related to the quality of baked tobacco middle leaves in four planting sites.

Sites	Total sugar (%)	Reducing sugar (%)	Total nitrogen (%)	Total alkaloids (%)
CX	43.82 ± 1.520 aA	26.61 ± 1.490 aAB	1.51 ± 0.080 cB	1.99 ± 0.110 bAB
JS	26.48 ± 8.868 bB	16.84 ± 5.954 bBC	2.11 ± 0.240 bA	3.59 ± 1.100 aA
XY	47.31 ± 1.412 aA	30.86 ± 1.775 aA	1.12 ± 0.123 dB	1.07 ± 0.181 bB
ZY	23.23 ± 1.799 bB	14.65 ± 1.441 bC	2.58 ± 0.081 aA	3.49 ± 0.203 aA

Different capital and smaller alphabets indicate the significant difference level with *p*-value <0.01 or *p*-value <0.05, respectively.

### 3.3 The changes of metabolites in tobaccos at the four planting sites

**Figure 2:**
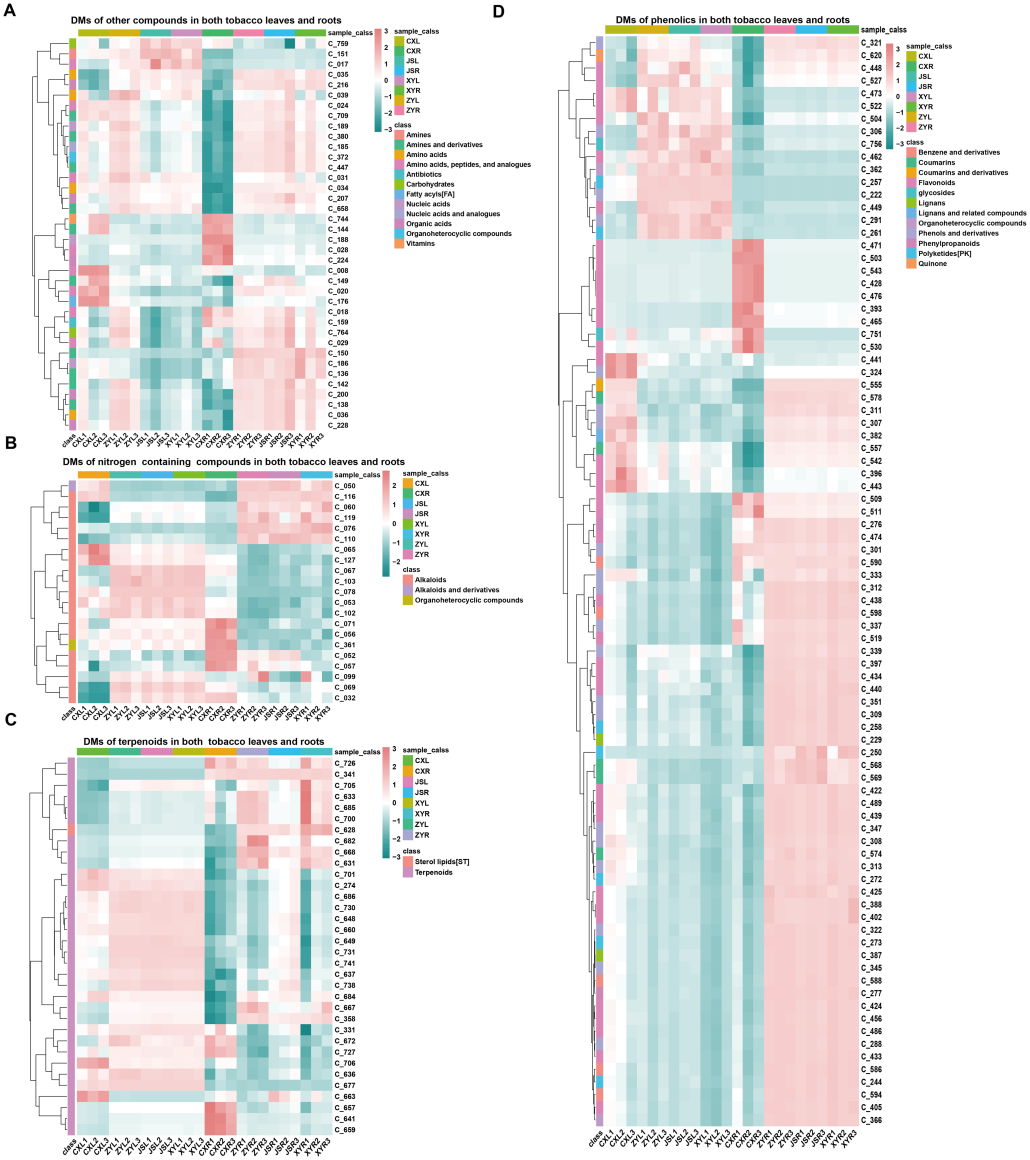
Differential metabolites found in both the leaves and roots. **(A)** other compounds, **(B)** nitrogen containing compounds, **(C)** terpenoids, and **(D)** phenolics. The name and structural information of metabolites (starting from C_) enlisted in Supplementary Table S3 and Supplementary Figure S3.

In order to understand the differences in metabolites accumulated in both fresh leaves and roots of tobacco at the four planting sites, both leaves and roots with three biological replications in the earlier flowering stage, with 1–2 fully opened flowers, were collected to conduct the analyses using LC-MS/MS. The results showed that a total of 770 metabolites, including 381 phenolics, 122 terpenoids, 95 nitrogen-containing compounds (NCCs), 65 amino acids (AAs), 34 organic acids (OAs), 19 lipids, 17 nucleic acids (NAs) and analogs, 10 glycosides, eight organic heterocyclic compounds (OHCs), six vitamins, five carbohydrates, four antibiotics, three imidazoles, and one indole-derived compound were detected (Supplementary Figure S1A; Supplementary Table S3). The “ropls” package of R (Thévenot et al., 2015) was used for PLS-DA analyses, and the results showed that the CX metabolites of both the leaves and roots were separated from all the other samples of the three planting sites (JS, XY, and ZY) in which the JS samples were slightly separated from the samples from ZY and XY (Supplementary Figures S1B,C). Filtering by log_2_ FC ≥1 and VIP >1, a number of differentially abundant metabolites (DMs) in the leaves of tobaccos from the four planting sites were obtained. The results showed that the leaves of CX (CXL) vs. leaves of XY (XYL) (CXL_vs_XYL), CXL vs. leaves of ZY (ZYL) (CXL_vs_ZYL), CXL vs. leaves of JS (JSL) (CXL_vs_JSL), JSL_vs_XYL, JSL_vs_ZYL, and XYL_vs_ZYL had 271, 290, 277, 17, 17, and 20 DMs, respectively (Supplementary Figure S2A), while the roots of CX (CXR) vs. roots of XY (XYR) (CXR_vs_XYR), CXR vs. roots of ZY (ZYR) (CXR_vs_ZYR), CXR vs. roots of JS (JSR) (CXR_vs_JSR), JSR_vs_XYR, JSR_vs_ZYR, and XYR_vs_ZYR had 348, 348, 354, 80, 77, and 52 DMs, respectively (Supplementary Figure S2C). Further analyses of the obtained DM set showed that in the leaves, 20 unique DMs [11 upregulated (Up) and 9 downregulated (Down)] between CXL_vs_JSL, 12 (7 Up and 5 Down) between CXL_vs_XYL, 19 (5 Up and 14 Down) between CXL_vs_ZYL, 7 (2 Up and 5 Down) between the XYL_vs_ZYL, and 3 (1 Up and 2 Down) between the JSL_vs_ZYL were found (Supplementary Figure S2B). While in roots, 19 unique DMs (5 Up and 14 Down) of CXR_vs_JSR, 15 (4 Up and 11 Down) of CXR_vs_XYR, 8 (4 Up and 4 Down) of the CXR_vs_ZYR, 16 (7 Up and 9 Down) of the JSR_vs_XYR, 9 (6 Up and 3 Down) of the JSR_vs_ZYR, and 17 (9 Up and 8 Down) of XYR_vs_ZYR were detected (Supplementary Figure S2D). It is interesting that even the soil conditions were not exactly the same among the four planting sites (Supplementary Table S1) and that significant DMs were found between CX and other three planting sites (JS, XY, and ZY), suggesting that water conditions and the previous year planted crops (e.g., rice planted at CX, while maize planted at JS, XY, and ZY in 2021) may have had more effects on the growth and development, resulting in changes of DMs in the planted tobaccos. Therefore, this study focused on the DMs between CX and other three sites (JS, XY, and ZY). The union set of DMs among CXL_vs_JSL, CXL_vs_XYL, and CXL_vs_ZYL were obtained with 331 DMs (named LDMU, showed in Supplementary Table S3), while the union set of DMs set among CXR_vs_JSR; CXR_vs_XYR and CXR_vs_ZYR resulted in 414 DMs (named RDMU, showed in Supplementary Table S3). The major metabolites out of 331 found in LDMU (with 265 up and 66 down) of leaves were SMs, including 165 phenolics, 54 terpenoids, and 44 NCCs (Supplementary Figures S3A,C,E,G), while in tobacco roots, 414 DMs found in RDMU (80 Up and 334 Down) were also mainly SMs, including 214 phenols and derivatives, 57 NCCs, and 65 terpenoids and their derivatives (Supplementary Figures S3B,D,F,H; Supplementary Table S3). Rhizosphere microbiota had close interactions with roots, so further sub-classification of SMs of the tobacco roots was conducted, resulting in flavonoids (98), terpenoids (56), alkaloids (46), polyketides (PK) (20), coumarins (18), benzene and derivatives (16), phenols and derivatives (14), lignans (10), phenylpropanoids (6), sterol lipids (ST) (5), and tannins (2) (Supplementary Table S3). Interestingly, the intersected LDMU and RDMU resulted in a set of 179 DMs (named C_179 showed in Supplementary Table S3) with phenolics (86), terpenoids (34), NCCs (21), and other metabolites (38) in which amines and derivatives (11), AAs and analogs (8), OAs (5), NAs (3), carbohydrates (2), OHCs (1), antibiotics (1), and vitamins (1) were detected. As SMs are the most accumulated compounds of C_179, in which 86 phenolics (containing 39 flavonoids, 20 phenols and derivatives, 8 polyketides (PKs), 5 benzene and derivatives, and 5 coumarins), 31 terpenoids, 19 alkaloids, and 8 PKs were found in the C_179 collections (Figure 2; Supplementary Figure S4). Furthermore, we focused on analyses of differentially abundant SMs of the C_179 set, and the results showed 6 phenolics (i.e., C_393|quercetin, C_465|homoeriodictyol, C_471|morin, C_503|hesperetin, C_509|myricetin, and C_530|narcissoside) which were consistently higher, while 11 phenolics (C_261|isoferulic acid, C_291|isoferulic acid, C_306|licochalcone C, C_321|byakangelicol, C_339|paeonol, C_527|glabrone, C_462|trilobatin, C_448|stilbin, C_449|isobavachin, C_504|diosmetin, C_620|purpurin) were consistently lower, accumulating in both roots and leaves of CX tobaccos than the other three sites. It was also found that 2 terpenoids (C_701|ginsenoside Re and C706| cantharidin) were higher, 3 terpenoids (i.e., C_628|picroside I, C_668| bergamottin, and C_726|lanosterol) were lower in both leaves and roots of CX, and there was only 1 alkaloid (i.e., C_071|dehydroglaucine) which was higher in both leaves and roots of CX than that of the other 3 sites (Figure 2; Supplementary Table S3; Supplementary Figure S4).

### 3.4 Tobacco rhizosphere microbiota and their gene diversities of the four planting sites

Microorganisms are crucial component of ecosystems and play an important role in shaping regional microenvironments. To determine the differences in rhizosphere microorganisms of tobacco at the four planting sites, the tobacco rhizosphere soils were collected for metagenomic analyses. An average of ~10 Gb clean data was obtained by sequencing using the Illumina platform for each DNA sample [with three replications (Supplementary Table S2)]. The assembling of megahits resulted in 60–160 Mb DNA sequences with 40–90 Kb lengths of contigs for each sample (Supplementary Table S4). For getting nearly complete individual genomes of bacteria, binning analyses were conducted, and the results showed that 422 bins were obtained (107 from CX, 107 from JS, 84 from XY, and 124 from ZY) (Supplementary Table S4). Further classification results showed that a total of 20 bins belonged to archaea, while 402 bins were detected as bacteria. The results of further sub-classification of archaea bins resulted in nine from CX, four from XY, four from JS, and three from ZY which belonged to the genus *Nitrososphaera*; however, CX, JS, and XY each had one unannotated bin. The taxonomic classification results of bacterial bins showed that the top three in phyla levels were Pseudomonadota (170), Acidobacteriota (54), and Gemmatimonadota (42); top three in class levels were Alphaproteobacteria (113), Gammaproteobacteria (45), and Gemmatimonadetes (42); top three in family levels were Sphingomonadaceae (91), Gemmatimonadaceae (29), and Pyrinomonadaceae (23); and top three in genus levels were *Sphingomicrobium* (79), *Pyrinomonas* (20), and *Nannochloropsis*|SCGC-AG-212-J23 (18) (Supplementary Table S4; Supplementary Figures S5A,B). Furthermore, different bin analyses among the four planting sites showed that, in the class level, the most abundant bins belonged to Alphaproteobacteria with JS (34) > CX (32) > ZY (30) > XY (17), while in the genus level, the most abundant bins belonged to *Sphingomicrobium* with ZY (23) = CX (23) > JS (21) > XY (11). Compared with the other sites, in class level, bins belonging to Blastocatellia, Vicinamibacteria, Limnocylindria, and Anaerolineae were higher and bins belonging to Gammaproteobacteria and Gemmatimonadetes were lower in CX than in the other three sites. At the genus level, PSRF01 and UBA12294 were higher and SCGC-AG-212-J23 were lower in CX than the other three sites (Supplementary Figures S5 C–J; Supplementary Table S4). For getting the Hbins, the dRep software was used to filter bins with standard of DNA length >50 Kb, integrity >75%, and contamination <25%, resulting in 13 Hbins with 5 in CX, 4 in JS, 1 in XY, and 3 in ZY. These Hbins were further identified into 2 archaea (1 in CX and 1 in JS) and 11 bacteria (4 in CX, 3 in JS, 1 in XY, and 3 in ZY) (Table 3). All Hbins could be annotated to the family level, while 10 of them could be annotated at the genus level; however, none of the bins can be annotated at the species level. The results of Hbins annotation suggest that these organisms may be new species which need further evidence, while mining the genes of these Hbins will be a new useful source for agricultural and industrial purposes. The results of different Hbins at the four different sites showed that two Hbins of Nitrososphaeracea each found in CX and JS belong to archaea, and no Hbins belong to archaea found in sites of XY and ZY. The CX site detected four bacterial Hbins, which were annotated as three Acidobacteriota and one Chloroflexota, three bacteria Hbins were found at the JS site annotated as two Gemmatimonadota and one Acidobacteriota, and one bacterium Hbins was found at the XY site annotated as Gemmatimonadota; however, three Hbins of bacteria were detected at the ZY site annotated as two Pseudomonadota and one Chloroflexota (Table 3; Supplementary Table S4).

**Table 3:** The information of 13 high quality bins.

Bin ID	Completeness	Contamination	N50	N90	Size	GC percent	Annotation classification
CX_B004	85.13	13.06	3,468	1,167	3,131,466	0.7000	d_B;p_Chloroflexota;c_Limnocylindria;o_QHBO01;f_QHBO01;g_;s_
CX_A009	84.73	20.6	1790	1,094	2,657,159	0.3572	d_A;p_Thermoproteota;c_Nitrososphaeria;o_Nitrososphaerales;f_Nitrososphaeraceae;g_TA-21;s_
CX_B003	99.15	7.88	10,421	2053	3,834,311	0.6655	d_B;p_Acidobacteriota;c_Thermoanaerobaculia;o_UBA5066;f_Gp7-AA6;g_Gp7-AA6;s_
CX_B021	75.62	15.91	2,239	1,125	4,539,899	0.5436	d_B;p_Acidobacteriota;c_Blastocatellia;o_Pyrinomonadales;f_Pyrinomonadaceae;g_PSRF01;s_
CX_B009	93.1	20.16	3,791	1,223	5,920,178	0.5387	d_B;p_Acidobacteriota;c_Blastocatellia;o_Pyrinomonadales;f_Pyrinomonadaceae;g_PSRF01;s_
JS_B005	90.75	20.77	5,418	1,538	3,749,936	0.6004	d_B;p_Gemmatimonadota;c_Gemmatimonadetes;o_Gemmatimonadales;f_Gemmatimonadaceae;g_UBA4720;s_
JS_B006	92.63	20.18	3,082	1,152	4,293,701	0.6641	d_B;p_Gemmatimonadota;c_Gemmatimonadetes;o_Gemmatimonadales;f_GWC2-71-9;g_;s_
JS_A002	99.93	4.43	27,083	1,465	2,325,507	0.3604	d_A;p_Thermoproteota;c_Nitrososphaeria;o_Nitrososphaerales;f_Nitrososphaeraceae;g_TA-21;s_
JS_B011	80.63	10.19	2,254	1,157	4,393,260	0.5758	d_B;p_Acidobacteriota;c_Blastocatellia;o_Pyrinomonadales;f_Pyrinomonadaceae;g_PSRF01;s_
XY_B005	86.13	18.39	4,009	1,246	3,446,043	0.6651	d_B;p_Gemmatimonadota;c_Gemmatimonadetes;o_Gemmatimonadales;f_GWC2-71-9;g_JAGGAG01;s_
ZY_B005-1	97.55	11.37	10,443	1,289	3,537,301	0.5834	d_B;p_Pseudomonadota;c_Alphaproteobacteria;o_Sphingomonadales;f_Sphingomonadaceae;g_Rhizorhapis;s_
ZY_B005-2	87.74	9.53	7,882	1861	5,779,225	0.5260	d_B;p_Chloroflexota;c_Anaerolineae;o_Anaerolineales;f_EnvOPS12;g_UBA12294;s_
ZY_B022	75.06	11.55	2,578	1,178	3,168,736	0.5463	d_B;p_Pseudomonadota;c_Gammaproteobacteria;o_Burkholderiales;f_Burkholderiaceae;g_;s_

d, p, o, c, f, g, and s: representing domain, phyla, order, class, family, genus, and species, respectively. d_A: archaea (A) & d_B: bacteria (B).

**Figure 3:**
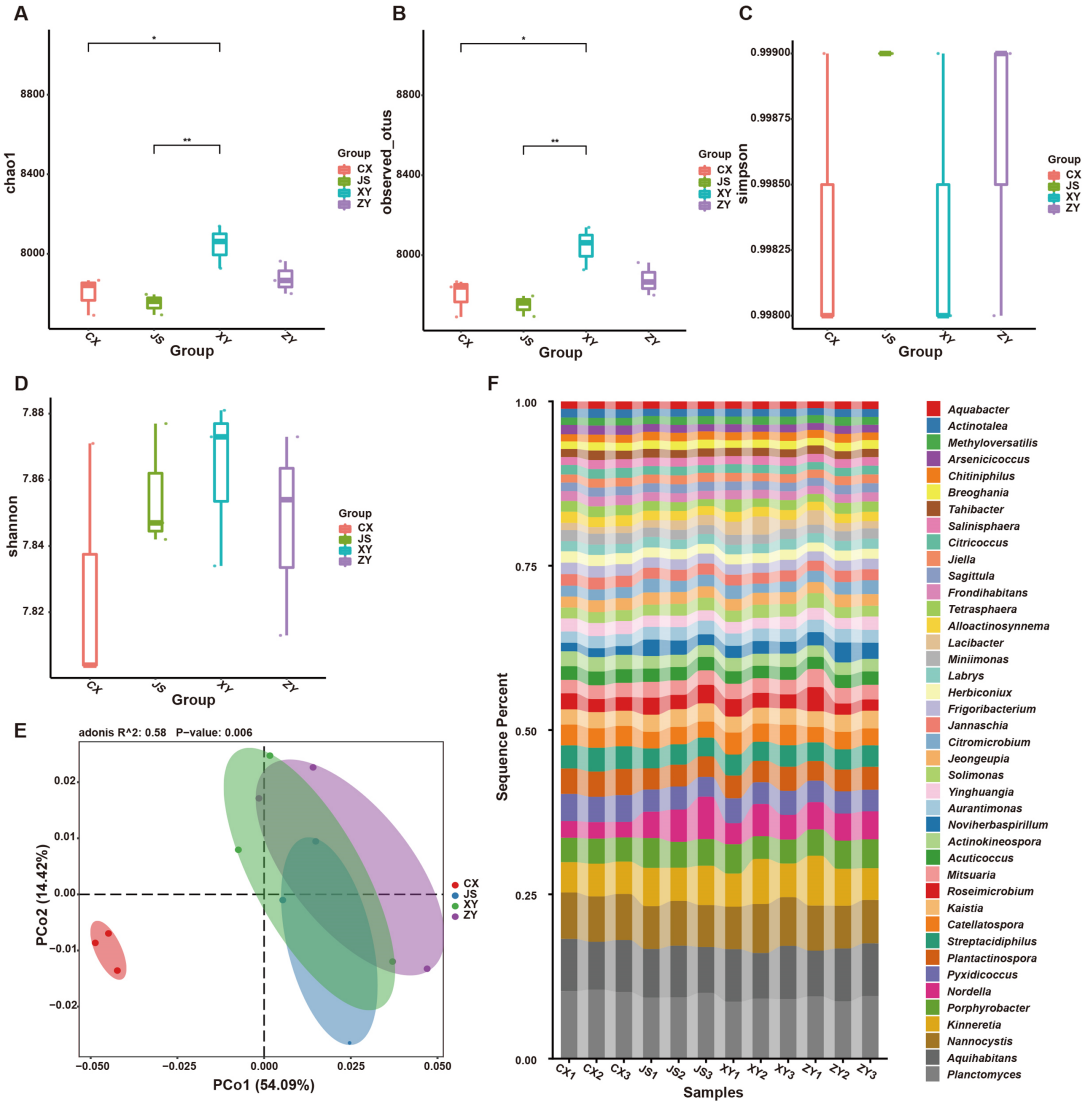
Abundance and diversity of tobacco rhizosphere microbiota in four planting sites. **(A)** Alpha diversity of rhizosphere microbiota of the Chao1 value, **(B)** obtained operational taxonomic unit (OTU) value of alpha diversity, **(C)** the Simpson value of alpha diversity, **(D)** Shannon values of alpha diversity, **(E)** beta diversity of rhizosphere microbiota shown by PCoA, and **(F)** heatmaps showed the abundance of top 41 microbiota at genera level of four planting sites.

**Figure 4:**
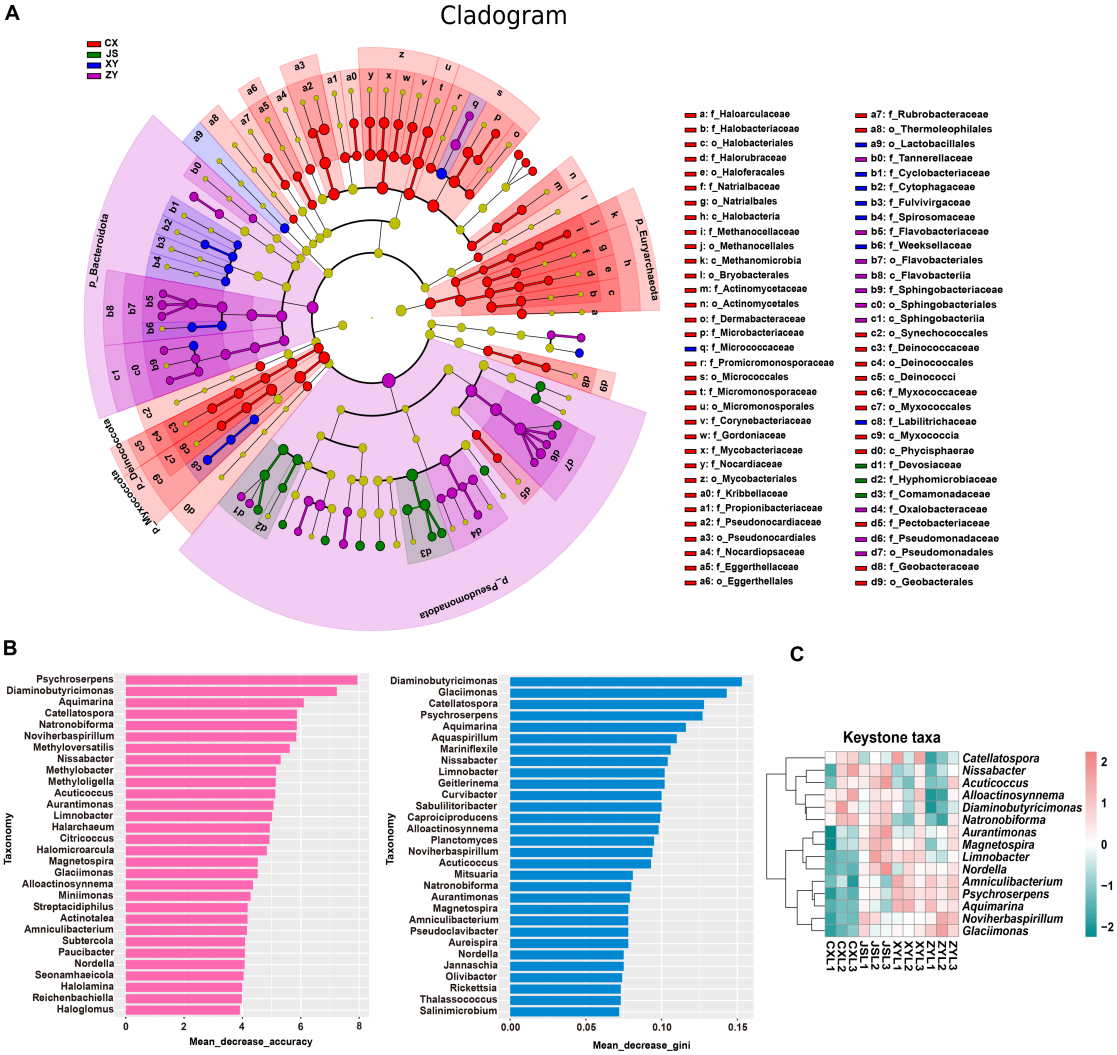
Differential abundance and key taxa screening of microbiota in four tobacco planting sites. **(A)** LEfSe diagram of rhizosphere microbiota, **(B)** identified important microbiota at the genera level based on the random forest algorithm, and **(C)** heatmap visualized the abundance of the screened key taxa at the genus level. Nodes in **(A)** represent relative abundance of identified microbiota as the larger node, the higher the relative abundance while the different colors of node represent the four different planting sites.

Furthermore, by using k-mer strategy analysis, a total of 8,479 species of microbiota, including three kingdoms (i.e., virus, bacteria and archaea), 58 phyla, 120 classes, 245 orders, 556 families, and 2,094 genera were detected. The analyses of both the alpha and beta diversity of organisms were performed, and the results showed that the number of Chao1 (Figure 3A) and obtained OTUs (Figure 3B) of microorganisms in the rhizosphere soil of tobacco in XY were higher than those in CX and JS, while there was no significant difference in the indexes of both Simpson (Figure 3C) and Shannon (Figure 3D) among the four planting sites. The beta diversity results showed that the CX samples were significantly separated from the samples of the other three sites (Figure 3E). Further evaluation of the microbiota abundance showed that *Planctomyces*, *Aquihabitans*, *Nannocystis*, *Kinneretia,* and *Porphyrobacter* were top five abundant genera of microbiota among the four planting sites (Figure 3F). Based on detected virus, bacteria, and archaea, top five class levels of virus were Naldaviricetes, Pokkesviricetes, Tectiliviricetes, Herviviricetes, and Caudoviricetes, top five family levels of virus were Poxviridae, Kyanoviridae, Tectiviridae, Herpesviridae, and Alloherpesviridae (Supplementary Figures S5M,N), top 5 classes of bacteria were Bacilli, Gammaproteobacteri, Betaproteobacteria, Actinomycetes, and Alphaproteobacteria, top 5 families of bacteria were Comamonadaceae, Nitrobacteraceae, Burkholderiaceae, Streptomycetaceae, and Sphingomonadaceae (Supplementary Figures S5K,L), top 5 classes of archaea were Thermoprotei, Thermococci, Methanomicrobia, Nitrososphaeria, and Halobacteria, and top 5 families of archaea were Halorubraceae, Nitrososphaeraceae, Halobacteriaceae, Haloarculaceae, and Natrialbaceae (Supplementary Figures S5O,P). It was interestingly found that there were no significant abundance differences in the top 41 microbiota at the genus level among the four planting sites. In these detected bacteria, viruses, and archaea, higher abundance at the class and family levels was shown (Supplementary Figures S5K–P). Although alpha diversity analyses initially showed differences in microbial species abundance among the four planting sites, to further understand the real key microbial taxa abundant differences in the four planting sites, LEfSe analyses were conducted and it was found that, at the phyla level, Euryarchaeota and Myxococcota were significantly enriched in the CX site, while Bacteroidota and Pseudomonas were enriched in XY, ZY, and JS. Additionally, under the phylum level [e.g., family (f), order (o) and class (c) level], families of Phycisphaerae, Pectobacteriaceae, Kribbellaceae, Propionibacteriaceae, Nocardiopsaceae, and Rubrobacteraceae and orders of Synechococcales, Geobacterales, Thermoleophilales, Eggerthellales, Pseudonocardiales, Mycobacteriales, Micromonosporales, Micrococcales, Actinomycetales, and Bryobacterales were enriched in the CX site; families of Micrococcaceae, Cyclobacteriaceae, Cytophagaceae, Fulvivirgaceae, Spirosomaceae, Flavobacteriaceae, and Weeksellaceae and order of Lactobacillales was enriched in the XY site; families of Devosiaceae, Hyphomicrobiaceae, and Comamonadaceae were enriched in JS, while families of Sphingobacteriaceae and Tannerellaceae; orders of Sphingobacteriales and Flavobacteriales and classes of Flavobacteriia and Sphingobacteriia were enriched microbiota in ZY site (Figure 4A). In order to further identify key genera among numerous detected microorganisms in the four planting sites, the analyses of the random forest algorithm were conducted, and the results of mean_decrease_accuracy (left of Figure 4B) and mean_decrease_gini (right of Figure 4B) were obtained. Filtered by mean_decrease_accuracy ≥4 and mean_decrease_gini ≥0.07, a total of 15 key microbial taxa in the genus level were selected. The results showed that the abundance of *Nissabacter*, *Acuticoccus*, *Alloactinosynnema*, *Diaminobutyricimonas,* and *Natronobiforma* was lower in both ZY and XY and higher in both CX and JS. The abundance of *Catellatospora* was higher in CX, JS, and XY, but lower in ZY. Interestingly, *Aurantimonas*, *Magnetospira*, *Limnobacter*, *Nordella*, *Amniculibacterium*, *Psychroserpens*, *Aquimarina*, *Noviherbaspirillum*, and *Glaciimonas* had lower abundance in CX than JS, XY, and ZY (Figure 4C, key_taxa sheet of Supplementary Table S4), suggesting these different microbiotas may correlate to DM accumulation in both leaves and roots of CX tobaccos compared to the JS, XY, and ZY planting sites.

**Figure 5:**
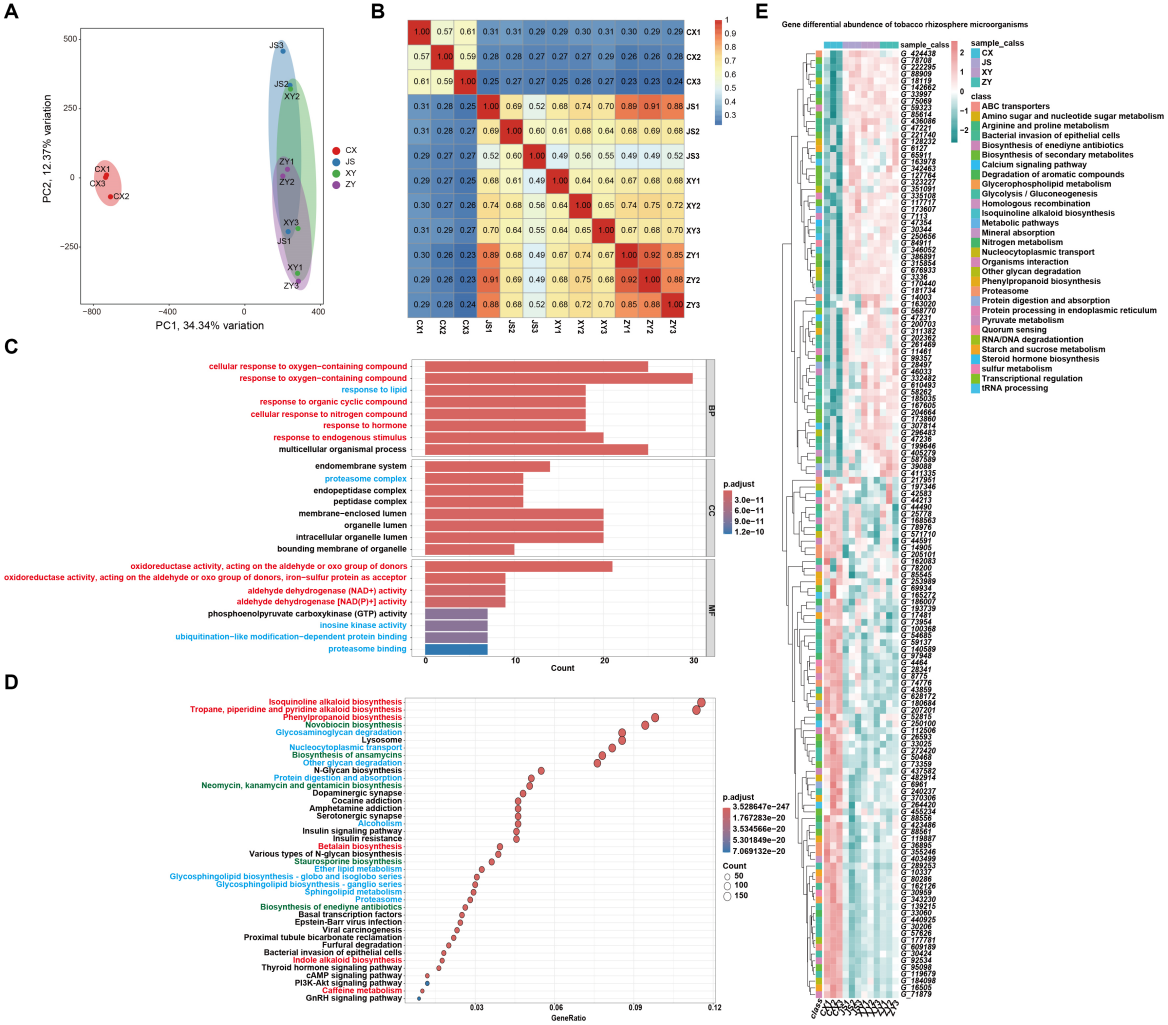
Metagenomic annotation, functional prediction, and analyses of gene abundance of rhizosphere microbiota. **(A)** PCA visualized gene abundance of rhizosphere microbiota in four planting sites, **(B)** heatmap of correlation of gene abundance with three replications of rhizosphere microbiota among four planting sites, **(C,D)** GO and KEGG enrichment analyses of different abundant genes (DAGs) among CX and other three sites, respectively (the red highlighted pathway is related to the synthesis of secondary metabolites, the blue highlighted pathway is related to the synthesis of primary metabolites, and the green highlighted pathway is related to antibiotics). **(E)** Heatmap visualization of the key 140 DAGs of CX as compared to other three planting sites (Gene information in E, start from G_, showed in Supplementary Table S5).

For analyses of the effects of microbiota coding genes on mutual interactions of tobacco and its rhizosphere microbiome, a total of 1,587,213 CDSs were obtained from ~1.4 Gb assembled microbiota DNA in all planting sites (Supplementary Table S5). After removing the redundant CDSs, a total of 851,209 CDSs (named as Unigenes) were obtained. The microbiota gene abundant levels were evaluated by mapping the fastq reads to CDSs, and then the differentially abundant genes (DAGs) were analyzed followed by screening by absolute log_2_FC ≥1 and *p*-adjusted value (*p*_adj_) <0.05 as the standard. The PCA analyses of the gene abundance in the four planting sites showed that the CX soil samples were significantly separated from the other samples of the three planting sites which overlapped together (Figure 5A). The gene abundance correlation analyses of all samples also showed that CX had a lower correlation with JS, XY, and ZY in which higher correlation values were found within samples from same sites (Figure 5B), suggesting that CX samples were more different from the other three sites, while JS, XY, and ZY had similar abundant genes of microbiota. Therefore, DAGs of CX vs. other sites were focused. There were 212,459, 249,550, and 251,408 DAGs found between CX vs. JS, CX vs. XY, and CX vs. ZY, respectively. As so many DAGs were detected, the intersections of DAGs of CX vs. JS, CX vs. XY, and CX vs. ZY were conducted to obtain a total set of 156,447 genes. Then, further GO and KEGG enrichment analyses were conducted, and the results showed that DAGs enriched in the GO biological process (BP) were related to cellular response to oxygen-containing compounds, phytohormones, lipids, and metabolism of SMs such as OAs and nitrogen compounds, etc. The cellular components (CC) enriched in the terms of the membrane-related intracellular organelle lumen and their membrane transportation, while the molecular function (MF) enriched in terms of oxidoreductase activities related to oxidation or reduction of aldehyde/oxo groups or metabolism of phosphoenolpyruvate (PEP). The KEGG enriched terms were also related to SM metabolism such as alkaloids (including isoquinolines and indole alkaloids), phenylpropanoids, antibiotics (e.g., novobiocin, ansamycins, neomycin, kanamycin, gentamicin, and staurosporine) and glycosphingolipid biosynthesis, biological processes of oxygen-containing compounds, lipids, and nitrogen compounds (Figures 5C,D). These important DAGs related to secondary metabolic pathways and bacterial membrane transportation collected resulted in a set of 140 genes, in which the gene abundance levels were visualized (Figure 5E; Supplementary Table S5). Interestingly, both GO and KEGG enrichment analyses showed DAGs of rhizosphere microbiota enriched in secondary metabolism of phenolics and alkaloids of isoquinolines and indoles which use aromatic AAs as synthetic precursors. However, the enriched DAGs of microbiota were not annotated as enzyme genes for phenolics and alkaloid biosynthetic pathways, but were annotated as genes encoding the aromatic AAs synthetic pathway for their survival or for preparing precursors of the abovementioned SMs. The enriched DAGs of microbiota also found encoding oxidative enzymes for oxidation of simple phenols or flavonoids to form polymers of lignins and tannins or glucosyl hydrolase for hydrolyzing sugar moieties of SMs in tobacco root exudates, which may relate to detoxification of these compounds for their rhizosphere microbiota attachments and proliferation (sheet of 140_genes_with_path_anno in Supplementary Table S5). The 140-gene set was further used for CCA analyses of the correlations among microbiota and tobacco metabolites in both the leaves and roots of tobacco as well as planting environmental factors.

### 3.5 The correlations among planting environmental factors, leaf and root metabolites, rhizosphere microorganisms, and their gene abundances

**Figure 6:**
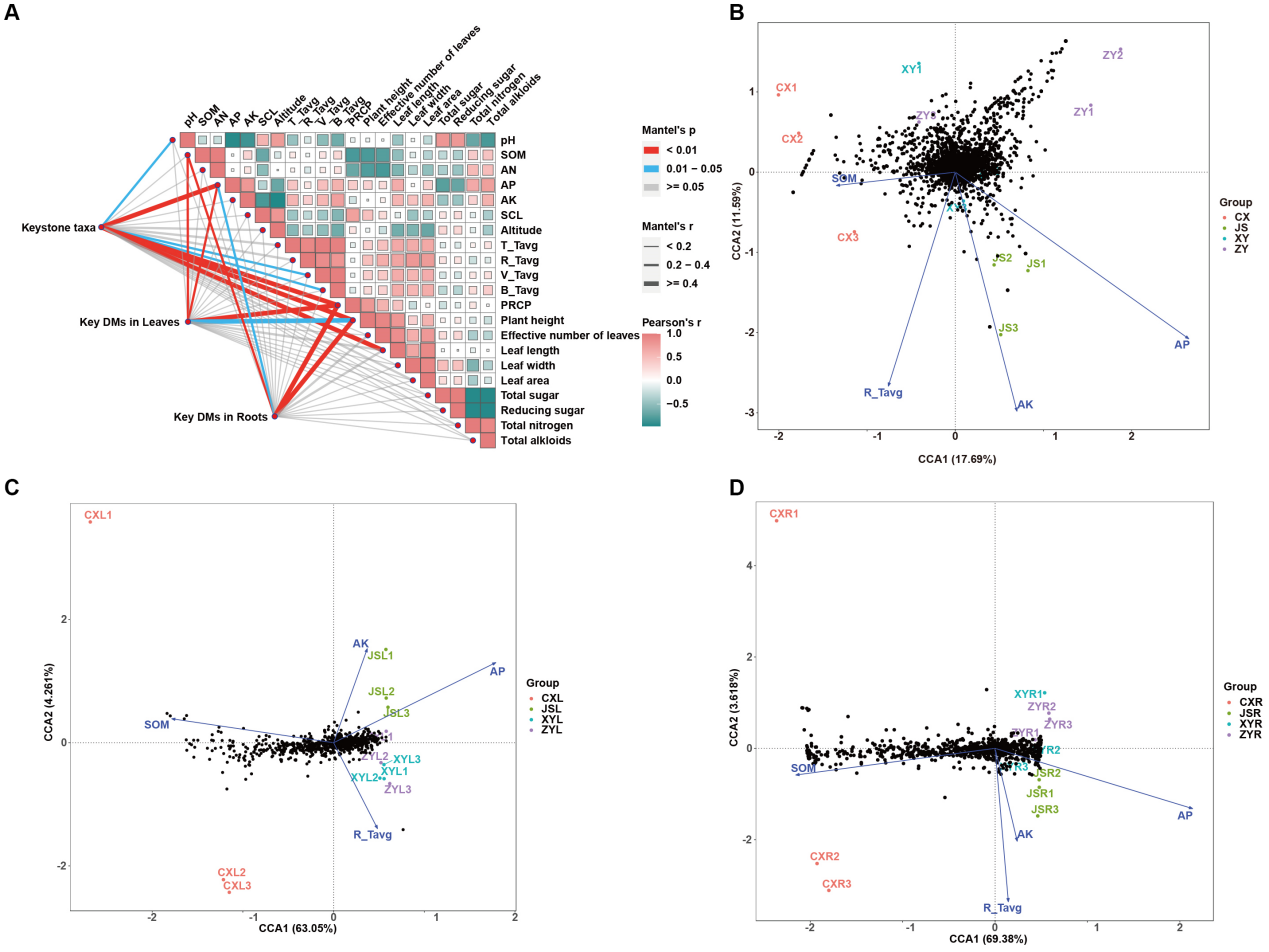
Correlation analyses among tobacco planting environments, agronomic traits, metabolites, and abundance of tobacco rhizosphere microbiota in four planting sites. **(A)** Mutual analyses of tobacco metabolite, rhizosphere microbiota, and tobacco planting environmental factors of four planting sites with Pearson’s correlation as well as Mantel test strategies. **(B)** Canonical correlation analyses (CCA) of rhizosphere microbiota correlated to the planting environmental factors, **(C)** CCA of metabolites in leaves correlated to the planting environmental factors, and **(D)** CCA of metabolites in roots correlated to the planting environmental factors. Key taxa and key DMs in both the leaves and roots are showed in sheet key_taxa of Supplementary Table S4 and sheet C_179 of Supplementary Table S3, respectively.

To determine the effects of planting environmental factors (soil pH, SOM, AN, AP, AK, SCL, altitude, average temperature of T periods (T_Tavg), average temperature of R periods (R_Tavg), average temperature of V periods (V_Tavg), average temperature of B periods (B_Tavg), and PREP in tobaccos planting sites) (Supplementary Table S1) on key DMs of tobacco leaves and roots (C_179 sheet of Supplementary Table S3) as well as key rhizosphere soil macrobiotic taxa (key_taxa sheet of Supplementary Table S4; Figure 4C), correlation analyses were conducted, followed by further confirmation with the CCA method. The results showed that soil factors of SOM and AP and climatic factors of PREP not only correlated to the agronomic traits of plants, including length, width, and area of leaves, but also to DMs in both the leaves and roots. The results also showed that the pH and AP and temperature of both V_Tavg and B_Tavg were significantly correlated to the abundances and diversities of microbiota of the tobacco rhizosphere, which resulted in significant changes in plant agronomic traits (Figure 6A; Supplementary Tables S6–S9). The results of further CCA analyses also support the correlation results obtained from the Mantel test, showing that the key environmental factors (e.g., AP, AK, R_Tavg, and SOM) were correlated to key taxa of rhizosphere microbiota of tobaccos with effects of AP > AK > R_Tavg > SOM. Among them, the key rhizosphere microorganisms in CX were negatively correlated with SOM as well as negatively correlated with AP (Figure 6B; Supplementary Tables S1, S4, S6–S8). The CCA results showed that DMs of both the leaves and roots also correlated with the abovementioned environmental factors. However, SOM, AP, AK, and R_Tavg were negatively correlated to DMs, in which SOM was more negatively correlated to DMs of roots than DMs of leaves. Interestingly, the Mantel test strategy used in this study did not show SOM correlation to key taxa of microbiota (Figure 6A); however, the method of CCA showed that SOM was negatively correlated to key taxa of microbiota, especially in the CX planting site. It is reasonable to postulate that SOM first affects the diversities of rhizosphere microbiota, and then the change in microorganisms finally shaped the metabolic pathways of tobacco, resulting in DM accumulation at the different planting sites, especially in roots of tobaccos. To verify the reliability of the above results, PLS-PM was used to construct a model to further analyze the correlation between the environmental factors (marked as ENV, e.g., altitude, temperature, and PREP), soil (e.g., pH, SOM, AN, AP, AK, and SCL), phenotypes (e.g., height, effective number, length, width, and area of tobacco leaves), key microbial taxa (marked as Key_taxa), and differential metabolites in roots (marked as Root_DMs). The resulting model showed that the goodness of fit index (Gof) of model was 0.6171, in which significant correlations between ENV and soil were detected with Estimate = −0.9944, and a significant correlation between key_taxa and Root_DMs was also obtained with Estimate = 0.8901 (Supplementary Figure S6). These results indicate that the environmental factors first influence the soil condition, resulting in changes of soil microbiota, then the changed microbiota shapes the Root_DMs, and finally influences the metabolic pathways, growth, and development of tobaccos.

Furthermore, root is the direct contacting organ of rhizosphere microbiota thus, further analyses of correlations among gene abundances of rhizosphere microbiotas, abundance of the key taxa in microbiota, and abundance of metabolites of tobacco roots were conducted. The results showed that *Amniculibacterium* was significantly correlated with 2 DAGs and 15 Root_DMs, *Aquimarina* (2 DAGs and 8 Root_DMs), *Aurantimonas* (1 DAG and 5 Root_DMs), *Glaciimonas* (19 DAGs and 20 Root_DMs), *Limnobacter* (6 DAGs and 3 Root_DMs), *Nordella* (3 DAGs and 14 Root_DMs), *Noviherbaspirillum* (11 DAGs and 18 Root_DMs), and *Psychroserpens* (39 DAGs and 73 Root_DMs), while *Alloactinosynnema*, *Diaminobutyricimonas,* and *Magnetospira* were associated with only one DAG without a significant correlation to any Root_DMs (Supplementary Figures S7A,B;Supplementary Table S6).

**Figure 7:**
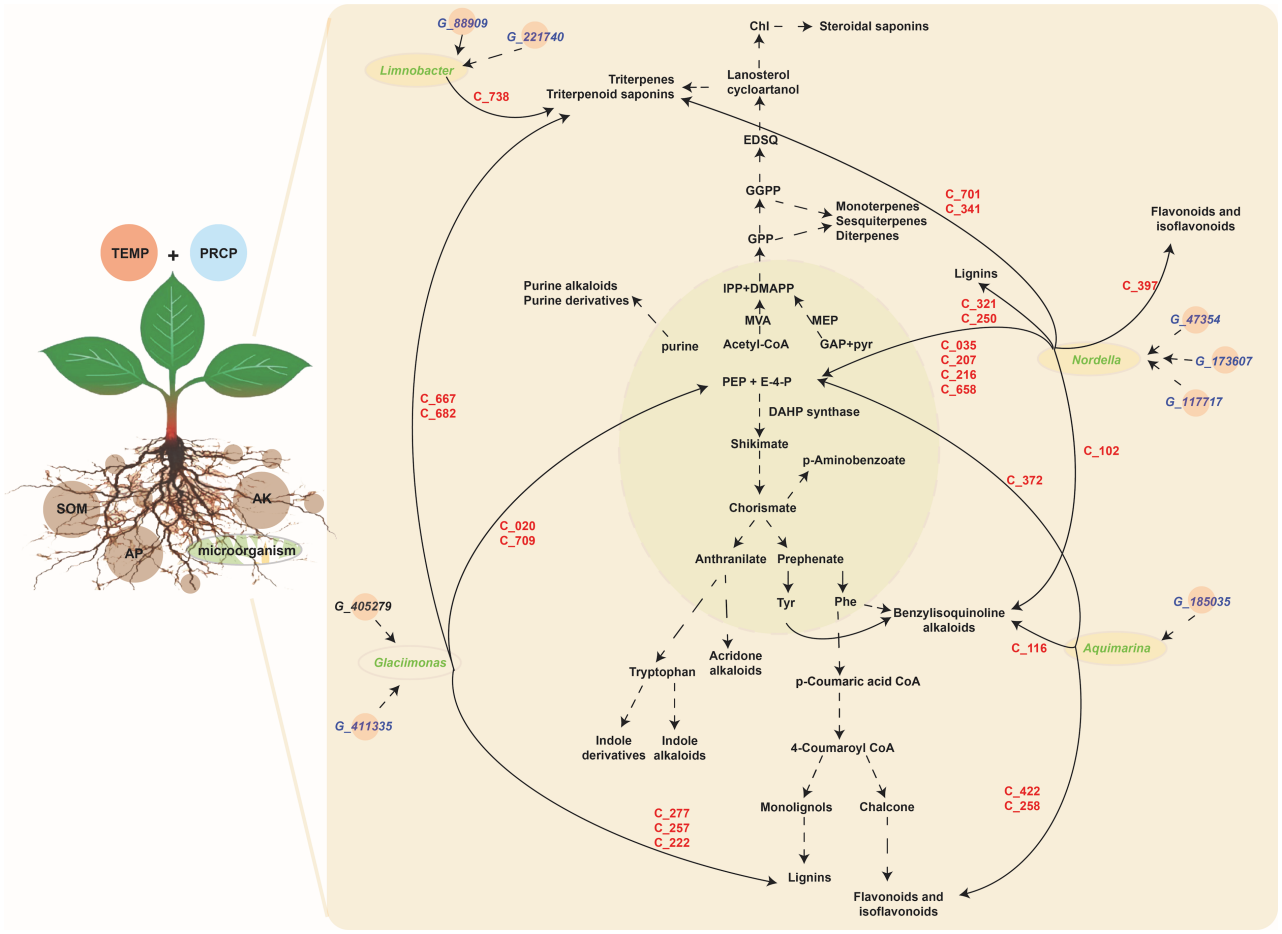
The metabolic pathway of DMs and correlation among the tobacco planting environmental factors, key taxa, key metabolite abundant of tobacco in roots, and metagenomic gene abundance of microbiota. Light green module demonstrates primary metabolism, and the light-brown module shows secondary metabolism, the gene information (starting from G_ in blue color) is shown in key_140_genes sheet of Supplementary Table S5, metabolite information (starting from C_ in red color) shown in key_metabolites sheet of Supplementary Table S3, while microbiota information on the genus level (in green color) shown in key_taxa sheet of Supplementary Table S4.

Based on the above correlation results, filtered by correlation value >0.87 and *p*-values <0.05, the key genera of rhizosphere microbiota (i.e., *Limnobacter*, *Aquimarina*, *Glaciimonas*, and *Nordella*) with higher abundance in JS, XY, and ZY than in CX were selected. *Limnobacter* is a more closely correlated to *G_88909* gene of microbiota, encoding the NADPH-dependent reductase converting L-glutamate 5-phosphate to L-glutamate 5-semialdehyde and *G_221740* encoding an enzyme of hexose group transferase and root metabolites of C738|7-O-ethylmorroniside, indicating that accumulation of *Limnobacter* may have more effect on terpenoid biosynthesis in tobacco roots. *Aquimarina* more correlated to *G_185035*, encoding bacterial glucokinase as well as metabolites of C_116|Sinomenine HCl, C_258|Isoliquiritin, C_372|5-Hydroxymethyl-2-furancarboxylic acid, and C_422|Hydroxygenkwanin, indicating that the genus of *Aquimarina* may have more effects on flavonoid biosynthesis of tobacco roots. *Glaciimonas* more correlated *G_411335* & *G_405279* encoding a protein for controlling the host cell meiotic calycle, and metabolites with the C_020|D-(-)-Penicillamine, C_222|p-Hydroxy-cinnamic, C_257|p-Coumaric acid, C_277|Ferulaldehyde, C_667|Neoandrographolide, C_682|Albiflorin acid and C_709|Secoxyloganin, indicating that *Glaciimonas* may have more effects on phenolics (e.g., lignin) and terpenoid biosynthesis. *Nordella* was more correlated with *G_47354*, encoding sodium/calcium co-transporter, *G_173607* encoding enzymes for electron transfer activities, and *G_117717* encoding an enzyme for conversion of oxaloacetate (OAA) to PEP and metabolites of C_102|Tetrandrine, C_035|D-Proline, C_207|Phosphonoacetic acid, C_216|Maleamic Acid, C_250|Cimifugin, C_321|Byakangelicol, C_341|Tanshinone IIA, C_397|Loureirin B, C_422|Hydroxygenkwanin, C_658|Gentiopicrin, and C_701|Ginsenoside Re, indicating that *Nordella* may have an effect on multiple metabolites of primary and secondary metabolism including sugars, simple phenolic acids, flavonoids, terpenoids, and alkaloids of benzylisoquinoline (Figure 7).

## 4 Discussion

### 4.1 The influence of the planting environment on the agronomic traits and metabolites of tobacco plants

The tobacco planting environment mainly includes temperature, PREP, altitude, and soil conditions. In this study, we found that during the growth period of tobacco, the PREP in CX was significantly lower than that in the other three sites, while temperature in JS was higher and soil pH was lower than in other three sites (Figure 1; Supplementary Table S1). The differences in climate and soil conditions resulted in significant phenotypic changes of tobacco including plant height, effective leaf number, leaf length, and quality-related chemical compositions of baked leaves. The agronomic traits of tobacco in JS were relatively good as length, width, and area of leaves; in addition, Mantel test results showed a significantly positive correlation between soil pH and TS contents, while a negative correlation with leaf length and TA contents was observed. There was a significantly negative correlation between altitude and leaf width and leaf area. Temperature was significantly positively correlated with leaf length, leaf width, and leaf area, while PREP was significantly positively correlated with plant height and effective leaf number, which is slightly different from Tang’s et al. (2020) research.

Tobacco metabolite profiles are important indicators for measuring plant environmental response. The results of the current study showed that the TS and RS contents in CX and ZY tobacco leaves were significantly higher, while the TN and TA contents were significantly lower (Table 2). The metabolome results showed that a total of 770 metabolites were detected in the four planting sites where CX had the highest number of DMs compared with the other three planting sites (Figure 2). Further analyses showed that the DMs in both tobacco leaf and root samples, with NCCs, phenolic compounds, and terpenoids were the most abundant (Supplementary Figure S3). This may be due to the activation of tobacco defense mechanisms in a stressful environment, resulted in changing the plant metabolic pathway for the syntheses and accumulation to increase the stress tolerance of CX tobaccos. Interestingly, most NCCs and phenolics were upregulated in the leaves, while terpenoids were downregulated in the leaves, and the expression of most DMs in the roots was opposite to that in the leaves of tobacco. There are many reports suggesting NCCs (most of them are alkaloids), phenolic compounds, and terpenoids as antibacterial and defensive to soil pathogens. Our result suggests that due to less PREP in CX, tobacco experienced drought stress, so plants made corresponding changes in physiological and metabolic pathways to accumulate different protective metabolites for improving drought tolerance for survival. The results were consistent at other three planting sites JS, XY, and ZY with relatively good PREP and temperature conditions, resulting in less number of DMs between tobacco samples of JS vs. XY, JS vs. ZY, and XY vs. ZY than CX vs. XY, CX vs. ZY, and CX vs. JS (Supplementary Table S3). Based on the correlation results by the Mantel test (Figure 6A) analysis and predicted the model by PLS-PM (Supplementary Figure S6), it can be concluded that environmental factors (especially SOM and PREP) and key taxa of microorganisms significantly affected the abundance of DMs in tobacco roots. This may be the main reason for the large number of significant DMs found between CX and the other three planting sites.

### 4.2 The shaping of rhizosphere microbiota by tobacco environmental factors

Rhizosphere microorganisms can have a significant impact on plants under environmental stress (de Vries et al., 2020). By using the metagenomic analysis strategies, the diversities of Yunnan tobacco rhizosphere were identified and found to have significant differences in the alpha diversity index by Chao1 (Figure 3A) and OTU (Figure 3B) in the four typical tobacco-producing sites. The results showed no significant differences in the Simpson (Figure 3C) and Shannon (Figure 3D) values of alpha diversities, indicating a significant difference in rhizosphere soil microbial abundance, but no significant difference of taxa in microbial community diversity among the four planting sites, suggesting the relative consistency of soil microbiota for uniform production of higher-quality tobacco leaves in Yunnan. Further LEfSe analyses indicate that microorganisms in phyla levels such as Euryarchaeota, Myxococcota, and Deinococcota were significantly enriched in CX, while Pseudomonadota and Bacteroidota were significantly enriched in the other three sites especially in ZY (Figure 4). While Methanomicrobia and Halobacteria of Euryarchaeota, Myxococcia of Myxococcota, and Deinococci of Deinococcota were enriched in the CX site, Oxalobacteraceae of Burkholderiales, Sphingobacteriales, and Flavobacteriaceae of Flavobacteriales in the ZY site, Weeksellaceae and Cytophagaceae of Bacteroidota in the XY site, Devosiaceae, Hyphomicrobiaceae, and Comamonadaceae of Pseudomonadota were enriched in JS sites (Figure 4).

Methanomicrobia is a strict anerobic microorganism whose metabolism relies on the formation of methane by reducing various substrates such as CO_2_, small methylated compounds, and acetate (Costa and Leigh, 2014); Halobacteria producing pigments of carotenoids captured solar radiation, increasing the temperature stress tolerance and reduced evaporation rate of plants in high salt environmental conditions (Grant et al., 1998). These microorganisms may also be reported to participate in processes such as hydrocarbon conversion, sulfur, nitrogen, iron cycling, and organic matter degradation (Baker et al., 2020). Myxococcia have strong decomposition functions in organic compounds (Reichenbach, 1999), while Deinococci, a type of microorganisms can resist stressful environments (e.g., strong radiation and drought) (Cox and Battista, 2005; Rainey Fred et al., 2005). Further key taxa in the genera level of microorganisms were selected by the analyses of the random forest algorithm, and abundance analyses of key genera showed that *Diaminobutyricimonas*, *Nissabacter*, *Alloactinosynnema,* and *Catellatospora* were significantly (*p*-value <0.05) higher in the CX site (Figures 4B,C), while *Psychroserpens*, *Aquimarina*, *Noviherbaspirillum*, *Aurantimonas*, *Glaciimonas*, *Amniculibacterium,* and *Nordella* were lower in CX than the other three planting sites. The significant enrichment of the above-mentioned microorganisms in CX may relate to the long-term drought resulted from low rainfalls and more solar radiations, a heavy rainfall in mid-August and last year’s paddy rice planting (Figure 1). Further GO and KEGG enrichment analysis of DAGs of tobacco rhizosphere microorganism results showed that the enriched genes were mainly classified in three categories: (1) the biosynthesis of SMs (e.g., phenylpropanoid, isoquinoline alkaloid, and indole alkaloid), (2) the biosynthesis of PMs (e.g., lipids, proteins, and carbohydrates), and (3) antibiotic synthesis (e.g., novobiocin, ansamycins, and neomycin). The relationship among plant roots, rhizosphere microorganisms, and microorganism gene expression is extremely complex as some studies suggest that root exudates can serve as signals for reshaping the root microbiota and helps alleviate abiotic stress in host plants by acting as chemotactic agents or nutritional source to rebuild the microbial communities. A study suggests that corn can benefit from arbuscular mycorrhiza fungi during drought, regulating water loss by reducing the expression of genes related to root water channel proteins (Gong et al., 2023). In addition, under drought stress, rhizosphere microorganisms can produce drought resistant-related phytohormones themselves (JAs and ABA) or induce production in their plant hosts. For example, in *A. thaliana*, genes upregulated under water deficit conditions may be highly correlated with *Pseudomonas chlororaphis* strain of O6 colonization, thereby activating the plant defense signaling pathways under drought stress, and the signals of such interactions are mostly SMs (Ali et al., 2017). In this study, when tobacco experienced drought stress due to low PREP under growth and development periods in the CX site, it stimulated the secretion of root metabolic compounds (especially phenolics, terpenoids, alkaloids, and AAs) (Figure 2). AAs, lipids, and other metabolites are usually used as carbon or nitrogen sources by rhizosphere microorganisms, while phenolics, terpenoids, and alkaloids with strong antibacterial activities may effectively change the microbial abundance, which may reduce harmful microorganisms and enrich beneficial microorganisms that may obtain genes encoding enzymes with the abilities to reduce the toxicity of SMs by redox/deamination, decarboxylation, and other modifications like glycosylation/de-glycosylation by plants and microbial coevolution. These beneficial organisms may also combat drought stress by clearing free radicals and mediating cell wall formation (Sanaullah et al., 2011). It is worth noting that among rhizosphere microbial species, microorganisms may compete for resources and space by secreting multiple antibiotics, which to some extent enhances plant stress resistance. High accumulations of phenolics, terpenoids, and alkaloids in CX tobaccos (Supplementary Figure S3) result in higher concentrations of SMs in roots exudates recruiting Myxococcota for degradation of SMs, paddy rice soil condition may recruit Methanomicrobia, while low moisture and higher radiation in the CX planting site (Figure 1) may result in Halobacteria and Deinococci recruiting rhizosphere of tobacco. The competition among species may result in species of genus including *Diaminobutyricimonas, Alloactinosynnema,* and *Catellatospora* which belongs to the class of Actinomycetes which can produce multiple antibiotics. As agronomic traits of other sites, especially XY and JS, were better than that of CX, so isolation of some strains of microbiota are abundant in XY and JS, and mining their genes may contribute for further and better cultivation of tobacco as well as other crops; simultaneously, bacterial synthetic communities composed of species of Myxococcaceae (Myxococcota), Halobacteriaceae (Halobacteria), Deinococcaceae (Deinococci), species in genus of *Diaminobutyricimonas* (Microbacteriaceae, Actinomycetes), species in genus of *Alloactinosynnema* (Pseudonocardiaceae, Actinomycetes), species of genus *Nissabacter* (Pectobacteriaceae, Gammaproteobacteria), and species in genus of *Catellatospora* (Micromonosporaceae, Actinomycetes) which were abundant at CX site (Figure 4) may enhance crop stress tolerance especially in drought conditions in Yunnan province of China.

## 5 Conclusion

The study screened 15 key taxa of rhizosphere microorganisms at the genera level, 140 important differentially abundant genes of microbiota were related more to secondary metabolism and facilitating bacteria membrane transferring of metabolites and 179 important DMs in the four planting sites of Yunnan, and the study also showed that temperature, PREP, soil pH, SOM, and altitude have significant impacts on the growth and development of “Yun87,” while PREP and SOM were the most critical environmental factors, which can significantly affect metabolites of tobacco leaves and roots, and soil rhizosphere microorganisms special bacteria such as *Diaminobutyricimonas*, *Nissabacter*, *Alloactinosynnema,* and *Catellatospora*. This research may promote the production and application of microbial fertilizers and agents to assemble synthetic microbiota community or mining important microbial genes for better cultivation of tobacco as well as other crops especially in Yunnan, China.

## Data availability statement

The datasets presented in this study can be found in online repositories. The names of the repository/repositories and accession number(s) can be found in the article/Supplementary material.

## Author contributions

RL: Data curation, Investigation, Validation, Visualization, Writing – original draft. ZL: Data curation, Investigation, Visualization, Writing – original draft, Validation. WD: Formal analysis, Visualization, Writing – original draft. XD: Data curation, Investigation, Writing – review & editing. EM: Data curation, Investigation, Writing – review & editing. NM: Visualization, Writing – review & editing, Software. CL: Data curation, Methodology, Supervision, Writing – review & editing. SZ: Data curation, Investigation, Writing – review & editing. WT: Data curation, Investigation, Writing – review & editing. MZ: Data curation, Investigation, Writing – review & editing. JL: Conceptualization, Funding acquisition, Methodology, Project administration, Resources, Supervision, Writing – review & editing. ZM: Conceptualization, Funding acquisition, Methodology, Project administration, Resources, Supervision, Writing – review & editing.

## Footnotes

^1^https://www.genomics.cn/

^2^https://cloud.metware.cn/

^3^https://www.bioladder.cn/

^4^https://rdrr.io/cran/pheatmap/

^5^https://solgenomics.net/jbrowse_solgenomics

^6^https://github.com/radiantgenomics/cdhit

^7^http://eggnog-mapper.embl.de/

^8^https://www.uniprot.org/

^9^www.tianqi24.com
