# Supplementary material for: Integrated metabolomic and metagenomic strategies shed light on interactions among planting environments, rhizosphere microbiota, and metabolites of tobacco in Yunnan, China
Source: Front Microbiol. 2024 May 9;15:1386150. doi: 10.3389/fmicb.2024.1386150 (PMC11112021; doi:10.3389/fmicb.2024.1386150)
Supplement: Supplementary file 1 [file Data_Sheet_1.PDF]

STable 1: Soil conditions of four tobacco planting sites

Planting sites	pH	SOM(g/kg)	AN(mg/kg)	AP(mg/kg)	AK(mg/kg)	SCL(mg/kg)
CX	6.8	34.6	143.3	11.5	175.2	16.1
JS	5.0	25.6	100.0	37.8	254.0	10.0
XY	7.1	11.1	42.3	16.7	145.0	50.0
ZY	6.0	26.7	128.3	26.1	160.0	42.7

STable 2: Statistics of metagenomic clean reads

Sample Name	Old_sample_id	Duplication	> Q30	Mb Q30 bases	GC	PF
CX1	T22CBST1A_1	0.50%	100.00%	10347.2	62.40%	100.00%
CX2	T22CBST2A_1	0.70%	100.00%	12047.1	62.40%	100.00%
CX3	T22CBST3A_1	0.60%	100.00%	12032.8	62.20%	100.00%
JX1	T22JBSCK1A_1	0.50%	100.00%	12012	61.20%	100.00%
JX2	T22JBSCK2A_1	0.50%	100.00%	11997.4	62.00%	100.00%
JX3	T22JBSCK3A_1	0.50%	100.00%	12000.9	62.50%	100.00%
XY1	T22XBSCK1A_1	0.50%	100.00%	12050.4	60.90%	100.00%
XY2	T22XBSCK2A_1	1.40%	100.00%	12024.1	59.90%	100.00%

XY3	T22XBSCK3A_1	0.90%	100.00%	12045.6	61.40%	100.00%
ZY1	T22ZBSCK1A_1	0.80%	100.00%	12050	59.50%	100.00%
ZY2	T22ZBSCK2A_1	0.70%	100.00%	12001.2	59.90%	100.00%
ZY3	T22ZBSCK3A_1	0.50%	100.00%	12028.4	61.60%	100.00%

STable 7: Results of first two dimensions of CCA between planting environmental factors and soil rhizosphere microbiota

Environment	CCA1	CCA2	R2	P-value
pH	-0.641304754	0.767286265	0.537766057	0.021
SOM	-0.97720988	-0.212275411	0.119406568	0.557
AN	-0.91107999	0.41222961	0.06167568	0.756
AP	0.77181658	-0.635845239	0.68164814	0.006
AK	0.138688849	-0.990336005	0.620448869	0.013
SSL	0.440250772	0.897874856	0.50353688	0.054
Altitude	-0.038361355	0.999263932	0.691642156	0.008
T_Tavg	-0.055545266	-0.99845617	0.617402469	0.019
R_Tavg	-0.315393984	-0.948960819	0.54859339	0.032
V_Tavg	0.03420481	-0.999414844	0.5627978	0.03
B_Tavg	0.044901795	-0.998991406	0.645110571	0.013
PRCP	0.966326812	0.257317882	0.544304552	0.037

STable 8: Results of first two dimensions CCA of planting environmental factors and metabolites in leaves

Environment	CCA1	CCA2	R2	P-value
pH	-0.854155006	-0.520018485	0.355772861	0.101
SOM	-0.998448517	-0.05568266	0.405609434	0.085
AN	-0.995403139	-0.095773644	0.311444479	0.179
AP	0.915703124	0.401855432	0.62593861	0.007
AK	0.624636001	0.780916043	0.184299885	0.378
SSL	0.936105269	-0.351719952	0.205232201	0.345
Altitude	-0.617858116	-0.786289609	0.171531477	0.401
T_Tavg	0.840067912	-0.542481246	0.084249694	0.699
R_Tavg	0.429926036	-0.902864112	0.122579231	0.535
V_Tavg	0.87937851	-0.476123342	0.09256218	0.636
B_Tavg	0.999817267	0.019116294	0.04125328	0.801
PRCP	0.992172255	0.124876804	0.801919584	0.01

Note: T_Tavg, R_Tavg, V_Tavg, M_Tavg are average temperature of transplanting period (T), rosette period (R), vigorous growing period (V) and budding & maturation period (B).

STable 9: Results of first two dimensions CCA of planting environmental factors and metabolites in roots

Environment	CCA1	CCA2	R^2	P-value
pH	-0.944457752	0.328632857	0.172263397	0.374
SOM	-0.993034271	-0.117825876	0.394314041	0.042
AN	-0.997775464	-0.066664255	0.26365147	0.171
AP	0.987740168	-0.156106888	0.400858656	0.04
AK	0.259527424	-0.965735738	0.106039962	0.553
SSL	0.932964692	0.359967894	0.301866225	0.118
Altitude	-0.171669082	0.985154671	0.111864532	0.53
T_Tavg	0.123545034	-0.992338967	0.33952961	0.107
R_Tavg	0.021525386	-0.999768302	0.283527176	0.179
V_Tavg	0.22195245	-0.975057491	0.323373737	0.139
B_Tavg	0.14571431	-0.98932671	0.257896364	0.225
PRCP	0.994100082	0.108466709	0.886119784	0.003

Note: T_Tavg, R_Tavg, V_Tavg, M_Tavg are average temperature of transplanting period (T), rosette period (R), vigorous growing period (V) and budding & maturation period (B).

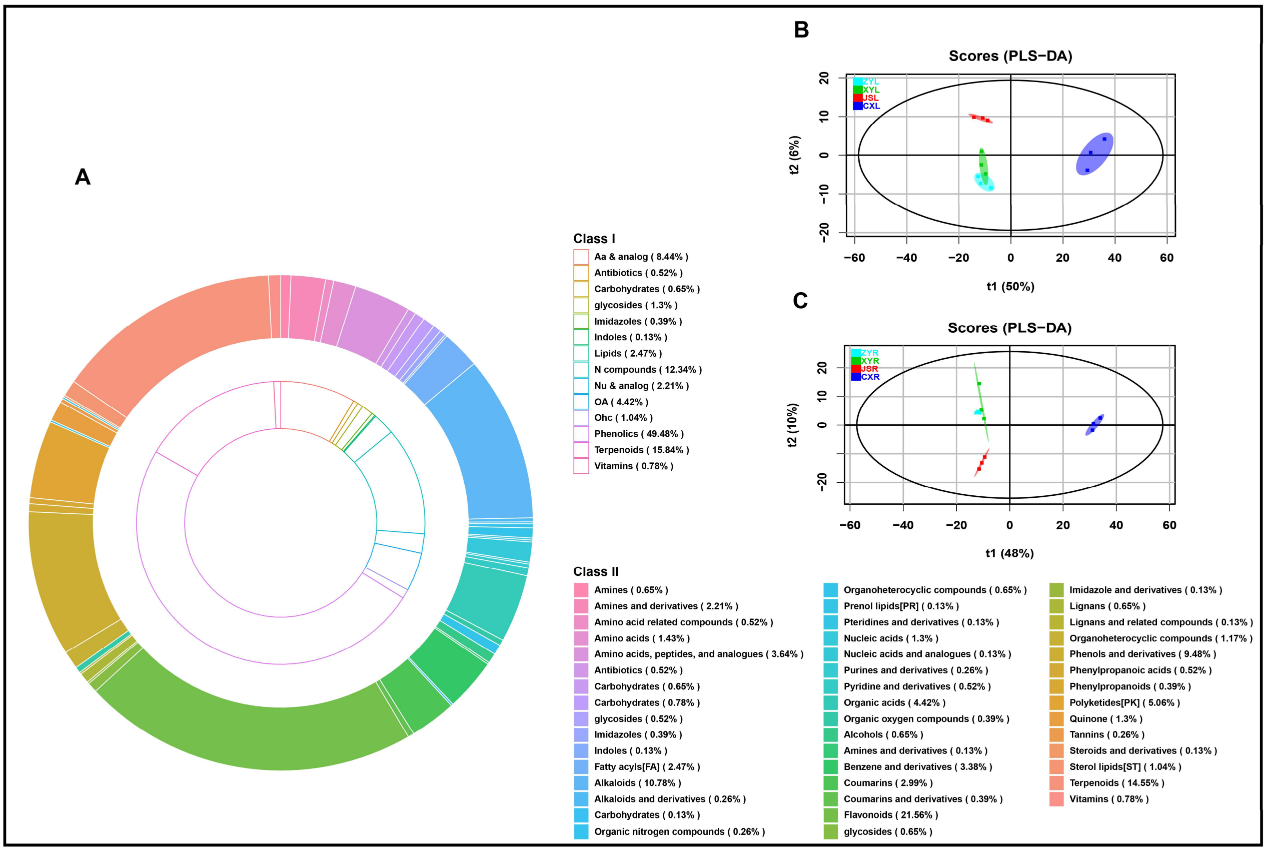

SFig 1: Classification and abundance analyses of detected metabolites in tobacco at four planting sites. (A) classification diagram of all detected 770 metabolites in both the leaves and roots of tobacco, (B) PLS-DA diagram of metabolites in tobacco leaves, (C) PLS-DA diagram of metabolites in tobacco roots.

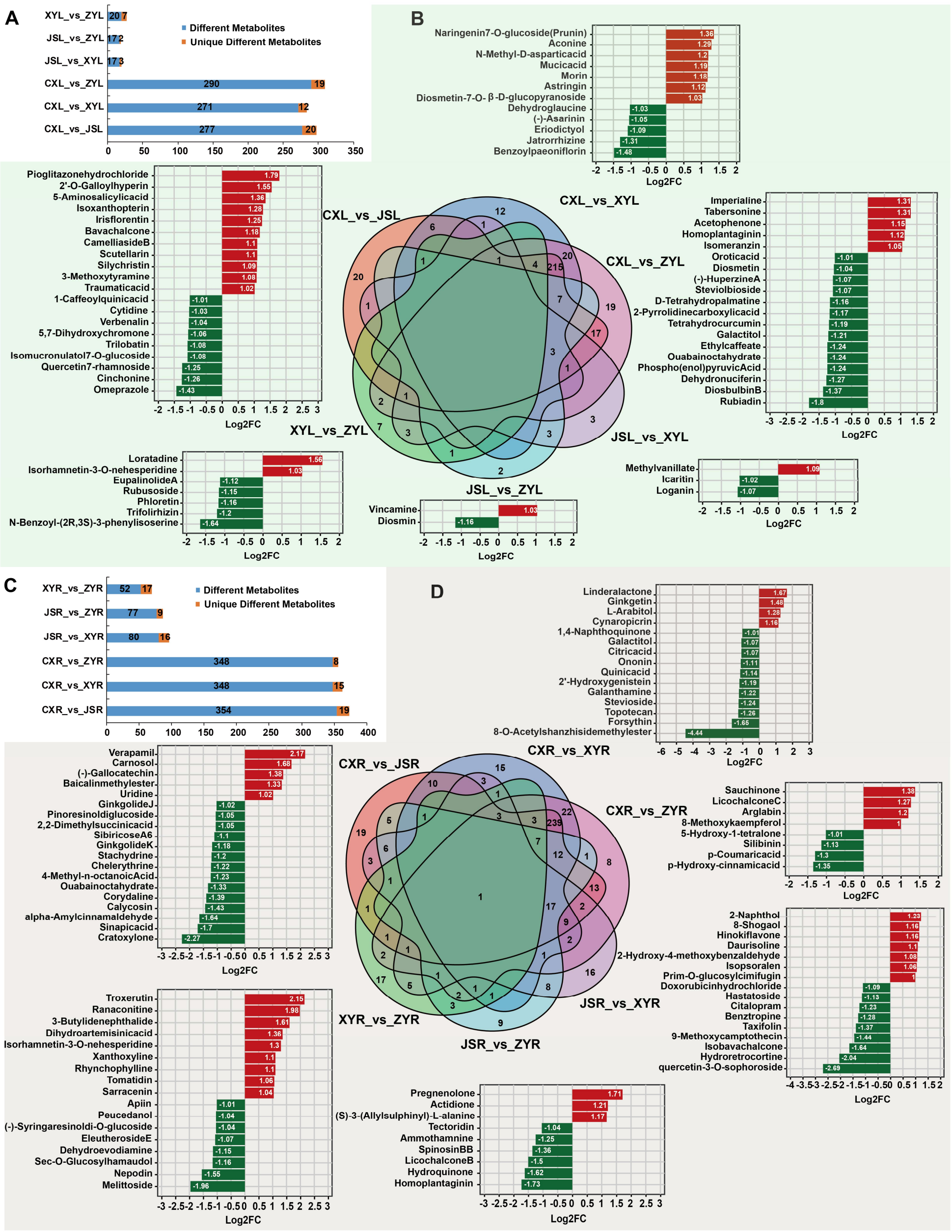

SFig 2: Different abundance of metabolites in tobacco among four planting sites. (A) tobacco leaf samples from 4 planting sites showed differences in the number of metabolites (DMs) and the number of unique differences in metabolites, (B) DMs in details of tobacco leaves and (C) number of DMs and unique DMs in tobacco root, (D) details of DMs in tobacco root samples.

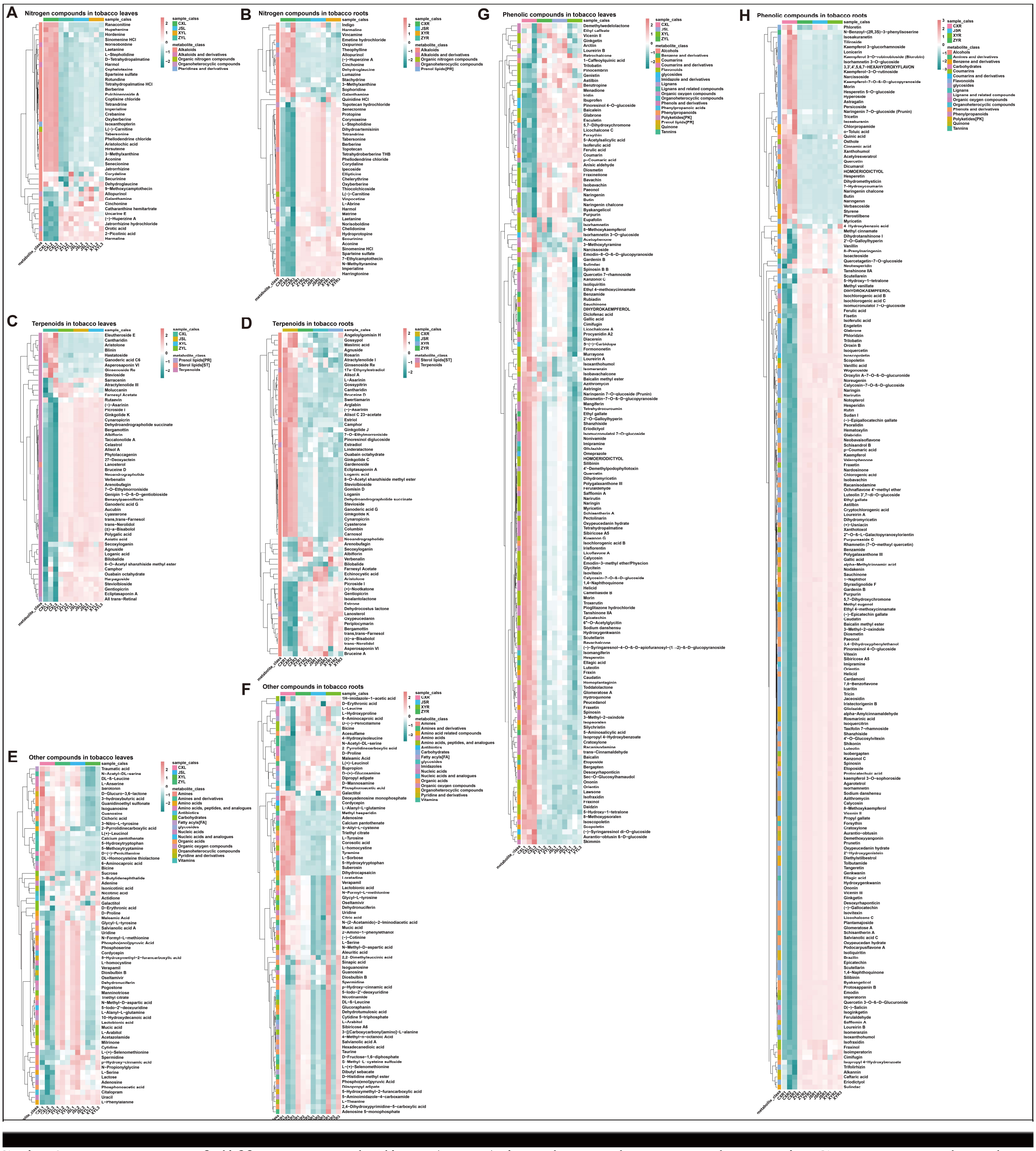

SFig 3: Heatmaps of different metabolites (DMs) in tobacco leaves and roots in CX compared to the other 3 planting sites. (A & B) the DMs abundance of nitrogen containing compounds in the leaves and roots respectively, (C & D) terpenoids, (E & F) other compounds and (G & H) phenolics in tobacco leaves and roots respectively.

Note: The detailed information showed in LDMU and RDMU sheets of STable3.

Other compounds

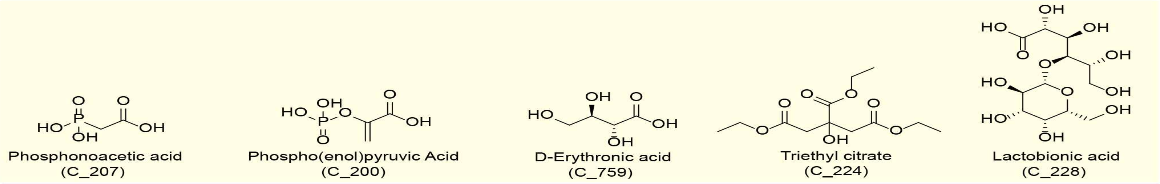

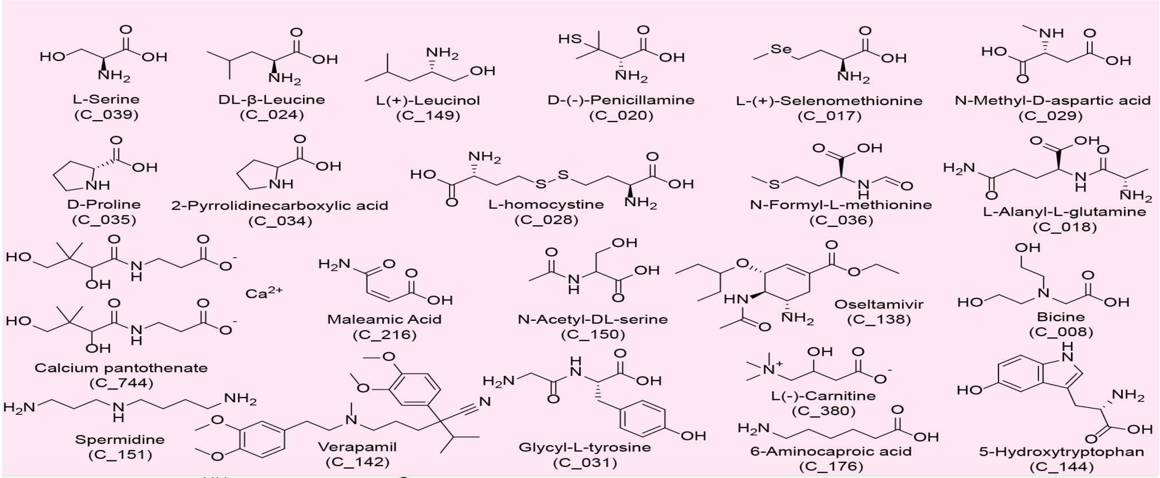

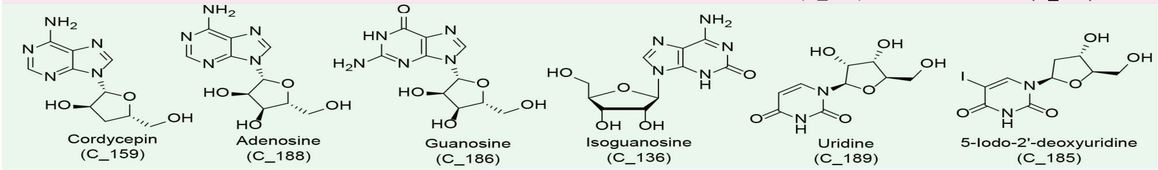

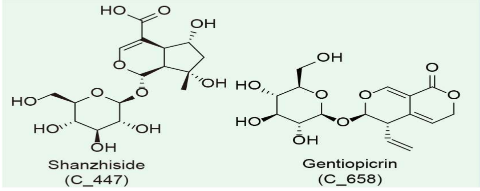

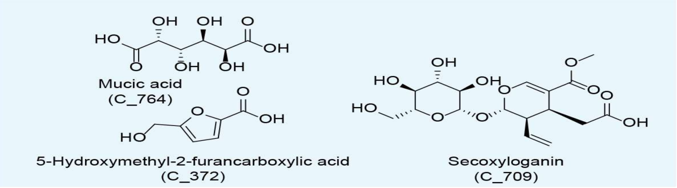

Nitrogen compounds

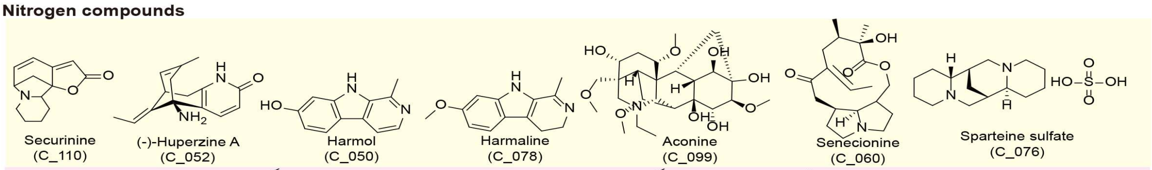

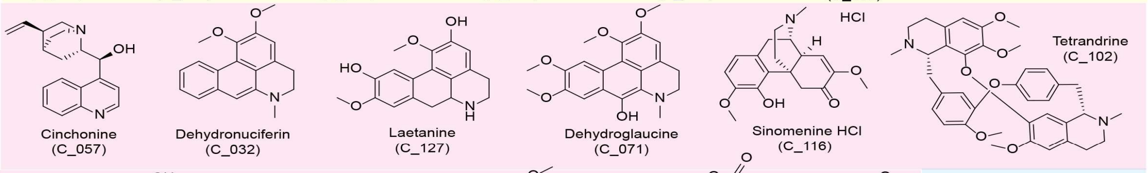

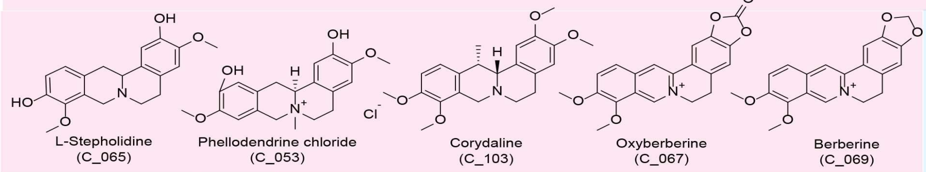

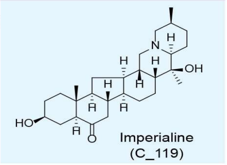

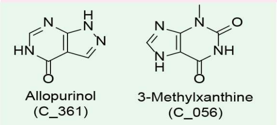

Continue of SFig4.

Terpenoid

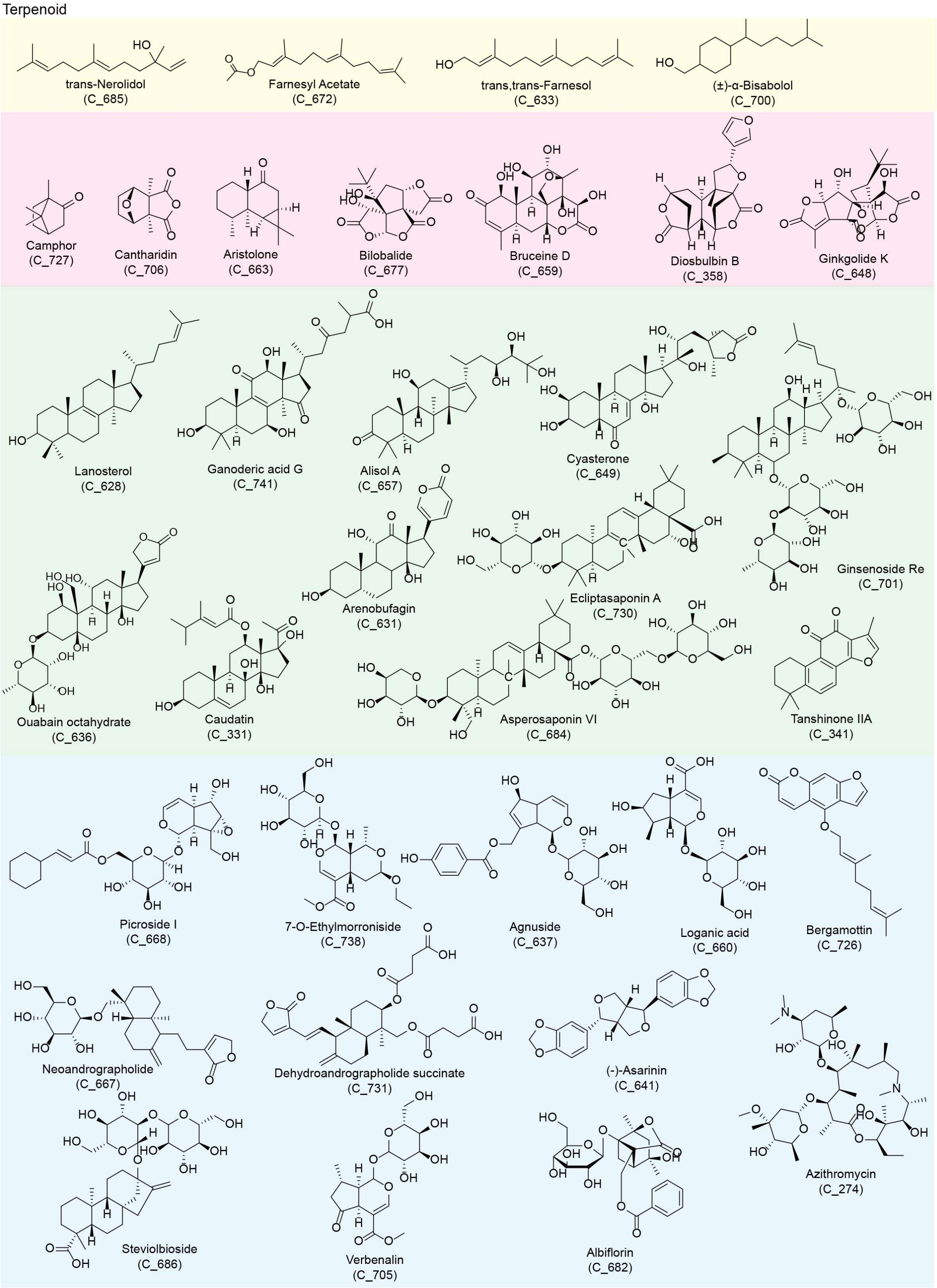

Continue of SFig4.

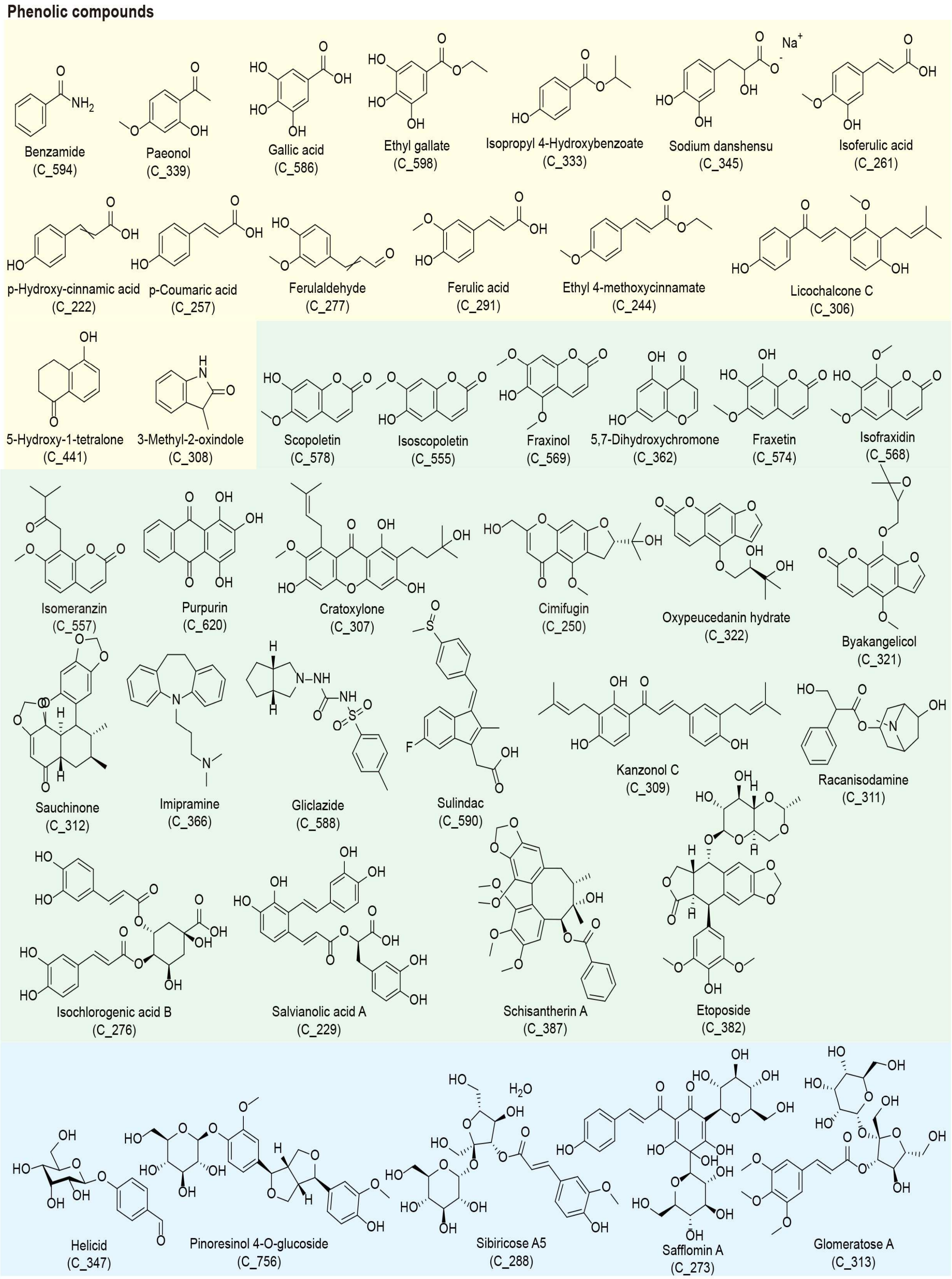

Continue of SFig4.

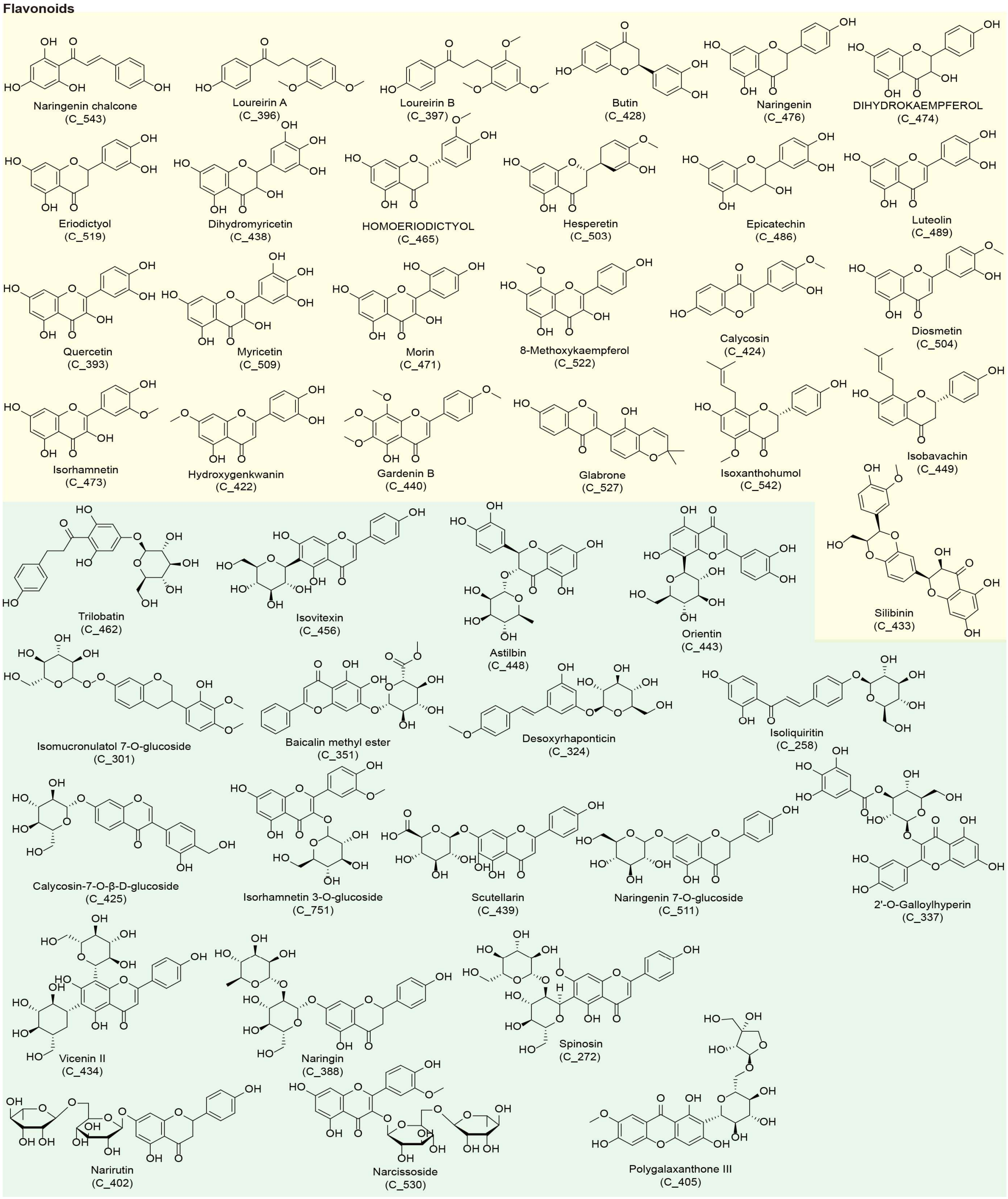

SFig 4: The DMs structures of C_179 with other compounds, nitrogen compounds, terpenoids, phenolic compounds and flavonoids between CX and the other 3 planting sites in both tobacco leaves and roots.

Note: The detailed abundant information and classification showed in C_179 sheet of STable3.

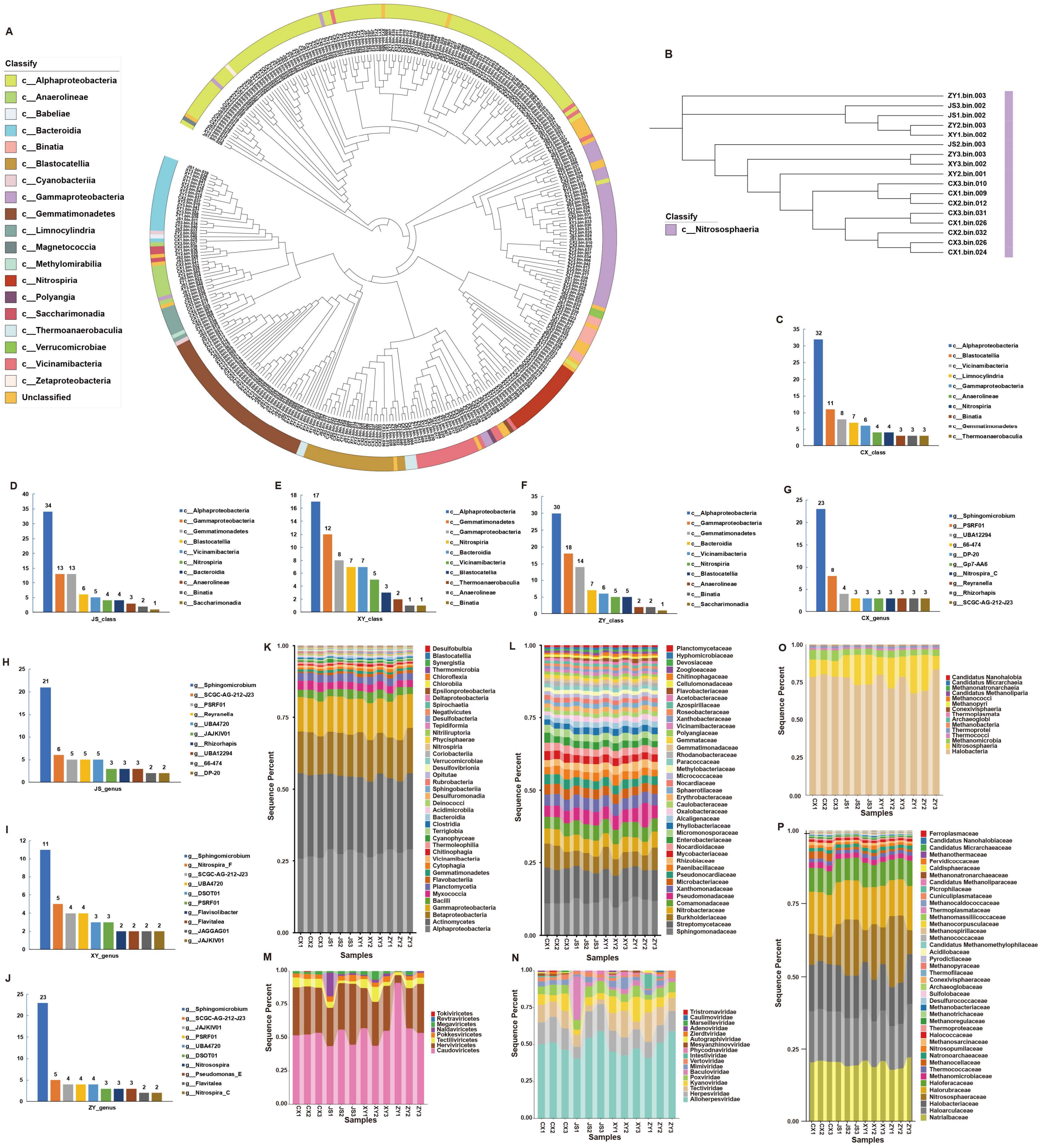

SFig5: The diversity and abundance of detected rhizosphere microbiota of tobacco.
(A) phylogenetic tree constructed based on the key marker genes of bins labelled as the class level of all bacteria, (B) phylogenetic tree of detected archaea bins labelled as class level, (C, D, E and F) the number of detected bins at class level of CX, JS, XY and ZY respectively while (G, H, I and J) the number of detected bins at genera level of CX, JS, XY and ZY respectively, (K & L) the abundance of bacteria in the top 41 at the class and family level respectively, (M & N) detected 8 viruses at the

class level and 17 viruses at the family level respectively, (O & P) detected 15 archaea at the class level, and 42 archaea at the family level respectively.
Note: The data in A to L was obtained by detection of megahit assemble following binning analyses with MaxBin2 and data in K to P was obtained by strategy of K-mer analyses by Kracken2 followed Braken2.

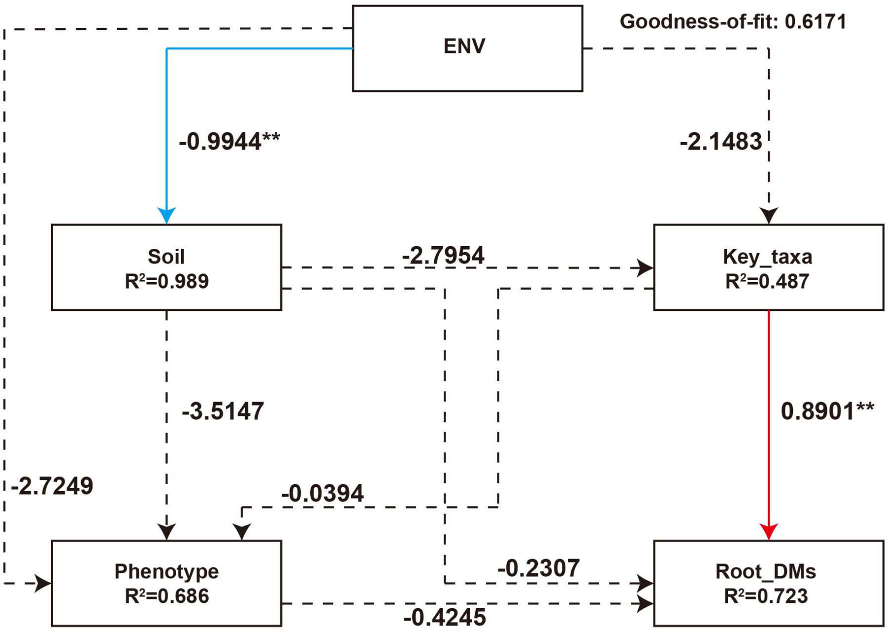

SFig6: Partial least squares path modeling based on environment, soil, phenotype, key_taxa and root_DMs. Solid lines show the significant and the dashed lines show no significant correlation. Whereas red color represents the positive and blue represents the negative correlation.

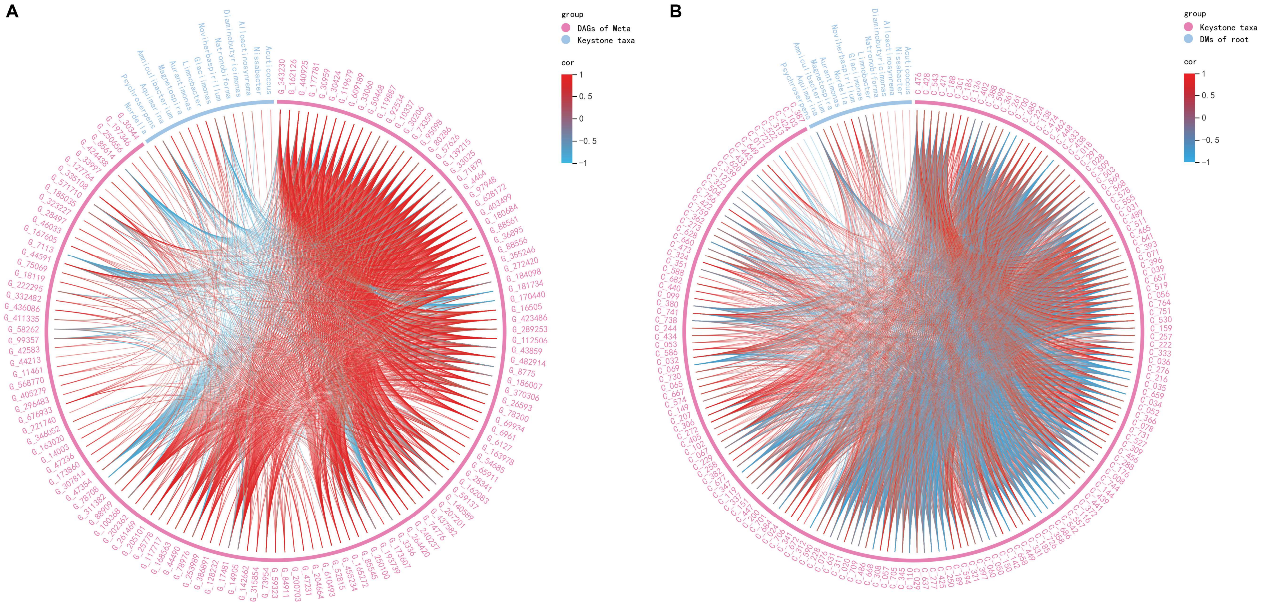

SFig7: Correlation analysis between rhizosphere microbiota and rhizosphere microbial different abundant genes (A) and between rhizosphere microbiota and root different metabolites (B).

Note: The abundance information of metabolites, key taxa and genes of rhizosphere microbiota are enlisted in C_179 sheet of STable3, Key_taxa sheet of STable4 and key_140_genes sheet of STable5.
